# A Single Dose of the DENV-1 Candidate Vaccine rDEN1Δ30 Is Strongly Immunogenic and Induces Resistance to a Second Dose in a Randomized Trial

**DOI:** 10.1371/journal.pntd.0001267

**Published:** 2011-08-02

**Authors:** Anna P. Durbin, Stephen S. Whitehead, Donna Shaffer, Dan Elwood, Kimberli Wanionek, Bhavin Thumar, Joseph E. Blaney, Brian R. Murphy, Alexander C. Schmidt

**Affiliations:** 1 Center for Immunization Research, Department of International Health, Johns Hopkins Bloomberg School of Public Health, Baltimore, Maryland, United States of America; 2 Laboratory of Infectious Diseases, National Institute of Allergy and Infectious Diseases, National Institutes of Health, Bethesda, Maryland, United States of America; Pediatric Dengue Vaccine Initiative, United States of America

## Abstract

Dengue is an emerging infectious disease that has become the most important arboviral infection worldwide. There are four serotypes of dengue virus, DENV-1, DENV-2, DENV-3, and DENV-4, each capable of causing the full spectrum of disease. rDEN1Δ30 is a live attenuated investigational vaccine for the prevention of DENV-1 illness and is also a component of an investigational tetravalent DENV vaccine currently in Phase I evaluation. A single subcutaneous dose of rDEN1Δ30 was previously shown to be safe and immunogenic in healthy adults. In the current randomized placebo-controlled trial, 60 healthy flavivirus-naive adults were randomized to receive 2 doses of rDEN1Δ30 (N = 50) or placebo (N = 10), either on study days 0 and 120 (cohort 1) or 0 and 180 (cohort 2). We sought to evaluate the safety and immunogenicity of this candidate vaccine in 50 additional vaccinees and to test whether the humoral immune response could be boosted by a second dose administered 4 or 6 months after the first dose. The first dose of vaccine was well tolerated, infected 47/50 vaccinees and induced seroconversion in 46/50 vaccinees. Irrespective of dosing interval, the second dose of vaccine was also well tolerated but did not induce any detectable viremia or ≥4-fold rise in serum neutralizing antibody titer.Only five subjects had an anamnestic antibody response detectable by ELISA following a second dose of vaccine, demonstrating that the vaccine induced sterilizing humoral immunity in most vaccinees for at least six months following primary vaccination.The promising safety and immunogenicity profile of this vaccine confirms its suitability for inclusion in a tetravalent dengue vaccine.

## Author Summary

Globally, dengue fever has become the most common clinically significant mosquito-transmitted viral illness. Dengue viruses exist as four serotypes, and increasingly several serotypes co-circulate in the same region. Infection with one serotype increases the risk of severe illness following infection with a second serotype. Therefore, any dengue virus vaccine needs to protect against all four serotypes. We and others are working to develop a live-attenuated tetravalent dengue vaccine that contains four monovalent vaccine viruses. Since two or more doses of such a vaccine are thought to be necessary for induction of long-lasting protective immunity, a feasible dose interval needs to be determined. Here, boosting with a second dose of a monovalent dengue type 1 (DENV-1) vaccine at four months or six months was compared in flavivirus-naïve healthy adult subjects with regard to safety, infectivity, and immunogenicity. We found that both doses of the vaccine were safe and well tolerated. While the first dose infected 92% of recipients, the second dose was neither infectious nor immunogenic, irrespective of the dose interval. These findings indicate that in most subjects, a single dose of this monovalent vaccine confers sterilizing humoral immunity against a second dose for at least six months.

## Introduction

Dengue fever has emerged as the world's most important mosquito-borne viral disease. Four antigenically distinct serotypes of dengue virus (DENV-1, DENV-2, DENV-3, and DENV-4) are transmitted by *Aedes aegypti* and *Ae. albopictus* mosquitoes, and the geographical spread of both mosquito vectors and the four viruses has led to an increased number of countries experiencing epidemic dengue fever [1]. In dengue endemic countries, hyperendemicity in many urban centers as well as focal outbreaks in rural areas are a major public health concern [2], [3]. Up to 3 billion people are at risk of infection in tropical and sub-tropical countries, and an estimated 50 – 100 million people develop dengue illness annually [1], [4], [5]. Although most dengue-infected individuals are treated on an outpatient basis, hundreds of thousands of hospitalizations and approximately 20,000 deaths per year are attributable to dengue. In many countries, children bear much of this disease burden [6].

The spectrum of DENV disease ranges from subclinical disease or undifferentiated febrile illness to classic dengue fever (DF) and to life-threatening dengue hemorrhagic fever/dengue shock syndrome (DHF/DSS). All four DENV serotypes are capable of causing the full spectrum of disease, although differences in virulence might exist [4], [7], [8]. Long-term homotypic immunity is induced by a single infection with DENV [9]; however, heterotypic protection is less durable [10], and pre-existing immunity to one DENV serotype has been identified as a risk factor for more severe disease upon secondary, heterotypic infection [8], [11], [12], [13]. Therefore, a DENV vaccine needs to induce long-lived protective immunity against all four DENV serotypes. In order to do so, more than one dose of a live attenuated tetravalent vaccine may be needed [14], [15], [16], [17], [18], [19], [20]. For example, two doses of the Mahidol/US Army/Pasteur PDK passaged tetravalent vaccine, given 180 days apart, were needed to achieve greater than 75% seroconversion against 3 or more serotypes [15]. Similarly, three dose of ChimeriVax are needed to induce a trivalent or tetravalent response in >80% of flavivirus-naive children and adults [14].

In preparation for tetravalent DENV vaccine studies, others have conducted two dose studies to evaluate the safety, infectivity and immunogenicity of a second dose of monovalent DENV vaccines [17]. Sun et al. reported 50% plaque reduction neutralization test (PRNT_50_) seroconversion rates between 46% and 100% following the first dose of their live attenuated monovalent DENV vaccines and limited seroconversion following a second dose given one or three months later [17].

The Laboratory of Infectious Diseases (LID) at the NIH has developed monovalent vaccines against all four DENV serotypes using a variety of strategies [21], [22], [23], [24], [25], [26].The NIH monovalent DENV-1-4 vaccines were found to be safe, highly attenuated, and immunogenic in healthy adult volunteers when given as a single dose of 10^3^ plaque-forming units (PFU) [22], [23], [24]. Dengue-like illness was not observed in the more than 500 vaccinees evaluated thus far, and vaccine-related serious adverse events (SAEs) did not occur. The most common reactogenicity events were an asymptomatic faint maculopapular rash over the trunk and proximal extremities and a transient neutropenia that lasted 2–3 days without associated clinical signs. Specifically, the rDEN1Δ30 vaccine, when evaluated as a single dose of 10^3^ PFU administered subcutaneously, caused low-level viremia (10 PFU/mL) in 9/20 vaccinees, starting around day 10 and lasting for approximately three days [22]. None of the volunteers developed a dengue-like illness, nor did any report vaccine-related reactogenicity that interfered with daily activities. Compared to placebo recipients, a significant increase in the occurrence of any solicited clinical sign or laboratory finding other than asymptomatic rash and neutropenia, was not observed in vaccinees. The vaccine induced a four-fold or greater rise in PRNT_60_ antibody titers in 95% of the vaccinees [22].

Here we present the results of a two-dose study of the monovalent rDEN1Δ30 vaccine in healthy adult volunteers. This study was designed to evaluate the safety and immunogenicity of a new lot of rDEN1Δ30 in 50 vaccinees in preparation for tetravalent vaccine trials and to determine a suitable interval for administration of a second dose of vaccine. The 4-month dosing interval was chosen because evaluation of tetravalent dengue vaccine formulations containing rDEN1Δ30 demonstrated that neutralizing antibody responses in rhesus macaques could be boosted with a second dose of vaccine given at 4 months (but not at 1 month) post dose one [10], [27]. The 6-month interval was chosen because clinical trials with ChimeriVax indicated that boosting at an interval longer than 4 months was preferable [14]. Two cohorts of 30 subjects were recruited to receive two doses of the rDEN1Δ30 vaccine or placebo with the second dose given either on Day 120 or on Day 180 after dose 1. While the first dose of DEN1Δ30 was safe, infectious, and immunogenic in 92% of the vaccinees, the second dose of the vaccine, given at either interval, did not cause detectable viremia and did not boost neutralizing antibody titers, indicating that a single dose of monovalent DEN1Δ30 vaccine induced sterilizing humoral immunity against a second dose of this vaccine for at least 6 months.

## Methods

### Ethics Statement

The study was performed under a NIAID-held investigational new drug application (BB-IND #11677) reviewed by the US Food and Drug Administration. The clinical protocol, consent form, and Investigators' Brochure were developed by Center for Immunization Research and National Institute of Allergy and Infectious Diseases (NIAID) investigators and were reviewed and approved by the NIAID Regulatory Compliance and Human Subjects Protection Branch (RCHSPB), the NIAID Data Safety Monitoring Board (DSMB), the Western Institutional Review Board (WIRB), and the Johns Hopkins University Institutional Biosafety Committee. Written informed consent was obtained from each volunteer in accordance with the Code of Federal Regulations (21 CFR 50) and International Conference on Harmonisation guidelines for Good Clinical Practice (ICH E6). The DSMB of the NIAID Division of Clinical Research reviewed all safety data at 6-month intervals.

### Trial Design and Study Setting

This single-institution parallel group Phase 1 trial was conducted as a randomized double-blind placebo-controlled study at the Center for Immunization Research (CIR) at the Johns Hopkins Bloomberg School of Public Health (JHSPH). Study subjects were enrolled between May 2007 and July 2008 under study protocol CIR-229, registered at ClinicalTrials.gov as Study NCT00473135. The study was designed to evaluate the safety of two doses of the rDEN1Δ30 vaccine, given at day 0 and again at day 120 or day 180, and to evaluate the virologic and serologic response to the vaccine after dose 1 and dose 2. Viremia was characterized by mean peak titer, day of onset, and duration. The serologic response was characterized by PRNT_60_ at study day 28 and 42 following both first and second vaccination.

Two cohorts were evaluated; cohort 1 subjects received vaccine or placebo at study day 0 and again at study day 120, subjects in cohort 2 received vaccine or placebo at study day 0 and again at study day 180. Within each cohort, subjects were randomized such that 25 would receive vaccine and 5 would receive placebo. A sample size of 25 vaccinees and 5 placebo recipients in each cohort was chosen based on our previous Phase I trial of this candidate DENV-1 vaccine [22] and to expand the safety evaluation of this vaccine. Subjects were randomized using a random number generator, by a member of the CIR who was not involved in the clinical or laboratory evaluation of the subjects. Subjects who had been enrolled could be replaced until study day 42 following first vaccination if they did not complete study day 28 or study day 42, or if their specimens were not usable in the immunological assessment at study day 42 due to protocol-defined reasons. If a volunteer was replaced, the safety data and viremia data for that subject, up to the point of replacement, were included in the analysis. Subjects retained the same treatment assignment as the subject they replaced and the treatment assignment remained blinded.

### Study Population

Healthy adult male and non-pregnant female volunteers were recruited from the Baltimore, Maryland and Washington DC metropolitan areas. Written informed consent was obtained from each volunteer in accordance with the Code of Federal Regulations (21 CFR 50) and International Conference on Harmonisation guidelines for Good Clinical Practice (ICH E6). Healthy volunteers 18 to 50 years of age were enrolled if they met the following eligibility criteria: normal findings during physical examination; negative for antibodies to all DENV serotypes, yellow fever virus, West Nile virus, St. Louis encephalitis virus; negative for hepatitis B and C; negative for HIV; normal values for complete blood count (CBC) with differential, aspartate aminotransferase (AST), alanine aminotransferase (ALT), creatinine, coagulation studies, and urinalysis. Additional safety-related exclusion criteria were also applied. Female volunteers were required to have a negative result on a urine pregnancy test at screening and on each vaccination day and were required to use a reliable method of contraception.

### Clinical Procedures and Evaluation

On study day 0, volunteers reported to the CIR outpatient clinic and were randomly assigned to receive either vaccine or placebo (vaccine diluent) given as a 0.5 mL subcutaneous injection. The appearance of the vaccine and placebo was identical. All study staff involved in the clinical and laboratory assessment of the subjects remained blinded to the treatment assignment until the study was unblinded at study day 42 following the second vaccination. Subjects were monitored for immediate adverse reactions for at least 30 minutes after vaccination. Each subject was given a digital thermometer and a diary card to record his/her oral temperature three times per day for 16 days. Clinical assessments were performed every other day through study day 16 and again on study days 21, 28 and 42 post-vaccination. During clinical visits, a medical provider performed a focused physical examination to evaluate for local reactogenicity (pain, erythema and swelling at the injection site) and systemic reactogenicity (lymphadenopathy, photophobia, rash, and hepatomegaly) and questioned subjects specifically about dengue-related symptoms (fever, headache, retro-orbital pain, nausea, photophobia, fatigue, myalgia, arthralgia, and rash). These signs and symptoms as well as the specific clinical laboratory studies described below were classified as solicited reactogenicity. In addition, subjects were questioned at each visit about other intercurrent illnesses. For each dose, blood was drawn at each assessment for detection of viremia through study day 16 post-vaccination and for antibody assay on study days 0, 28 and 42 post-vaccination. Prior to vaccination, baseline CBC with differential, ALT and/or AST and coagulation studies were obtained. After each vaccination, a CBC with differential was obtained every other day through study day 16. Coagulation studies were done every fourth day through study day 16 post-vaccination and again at day 28 post-vaccination. Serum for ALT testing was collected every other day from study day 4 through day 16 and again on study day 21 post-vaccination. Subjects returned to the clinic at study day 90 (cohort 1) or study day 150 (cohort 2) for repeat HIV, hepatitis B and hepatitis C testing and to assess for continued eligibility for second vaccination. Exclusion criteria for second vaccination included positive HIV-1 serology, active hepatitis B or hepatitis C infection, pregnancy, receipt of a live vaccine within 4 weeks prior to second vaccination or a killed vaccine within 2 weeks prior to second vaccination, use of immunosuppressive doses of corticosteroids (excluding topical or nasal) or immunosuppressive drugs within 30 days prior to second vaccination, receipt of an investigational agent in the 30 days prior to second vaccination, or history of severe allergic reaction or anaphylaxis associated with first vaccination. Clinical and laboratory assessments following the second vaccination were identical to the first vaccination throughout the 42-day follow-up period. All adverse events were graded for intensity and relationship to vaccine. Fever was determined by oral temperature recorded on two consecutive measurements 1 hour or more apart and was defined as grade 1 (38.0°C–38.6°C), grade 2 (38.7°C–39.1°C), or grade 3 (>39.1°C).

### Adverse Events

Adverse events were graded as mild (no effect on daily activity), moderate (required an intervention or interfered with daily activity), or severe (prevented daily activity). Abnormal hematology, coagulation and serum chemistry findings were also graded as mild, moderate, or severe, using standardized toxicity tables. A dengue-like syndrome was defined as infection associated with fever and ≥ 2 of the following symptoms: Grade 2 headache lasting 12 hours, Grade 2 photophobia lasting ≥12 hours, Grade 2 generalized myalgia lasting ≥12 hours. Grade 2 retro-orbital pain lasting ≥12 hours, or sustained or intermittent epistaxis lasting ≥24 hours. Dengue infection was defined as recovery of vaccine virus from the blood and/or seroconversion to DENV-1 as measured by 60% plaque-reduction neutralization titer (PRNT_60_). All adverse events and abnormal clinical findings were collected for the duration of the study and were followed to resolution. Serious adverse events were defined in accordance with 21 CFR312,32.

### Vaccine

rDEN1Δ30 is a recombinant, live attenuated virus derived from the DENV-1 Western Pacific (WP) wild-type virus by deletion of 30 nucleotides in the 3′ UTR [28]. The vaccine virus in the current clinical lot of this investigational vaccine (Lot DEN1#104A, Charles River Laboratories, Malvern PA) is identical at the amino acid level to that evaluated in an earlier phase I study (Lot DEN1#1) [22]. A cDNA clone was generated to match the sequence of the previously manufactured rDEN1Δ30 (LotDEN1#1) and vaccine virus was derived and manufactured in qualified Vero cells. The genome sequence of the vaccine virus in Lot DEN1#104A differed from that of Lot DEN1#1 at seven nucleotide positions, however all substitutions were translationally silent. Vaccine virus was stored at -80±15°C until use. Just prior to vaccination, vaccine virus was removed from the freezer and diluted to a concentration of 3.3 log_10_ PFU/mL with vaccine diluent (1X Leibovitz L-15 medium lacking phenol red). L-15 was also used as the placebo. The 1X Leibovitz L-15 medium was prepared from a qualified lot of 2X Leibovitz L-15 (Lonza, Walkersville, MD) mixed 1∶1 with sterile water for injection. Diluted vaccine was administered as a 0.5 mL subcutaneous injection within four hours of removal from the freezer . The virus titer of the diluted and undiluted vaccine was determined to confirm the potency of the vaccine.

### Virus quantitation and serologic assessment

Virus titers were determined by plaque assay after inoculation of undiluted or serial 10-fold dilutions of serum onto Vero cell monolayer cultures, as described previously [21]. The lower limit of virus detection in this assay is 0.5 PFU/mL. Antibody responses to DENV-1 virus were determined by PRNT_60_ as described previously [21], using DENV-1 (WP) as the target virus in the assay. For comparison of the PRNT_60_ values induced by the different vaccine lots, the day 42 samples collected during the previous clinical trial of rDEN1Δ30 were assayed in parallel with those collected during this trial. Seroconversion was defined as a ≥4-fold rise in serum neutralizing antibody to wild-type DENV-1 by study day 42 following each vaccination. Following first vaccination, this corresponded to a PRNT_60_ of ≥1∶20 as all subjects were flavivirus naïve (PRNT_60_<1∶5). Following second vaccination, the titer obtained on day 42 post-second vaccination (day 162 or day 222) was compared with the titer from Study Day 120 or Study Day 180. An IgG ELISA against whole virus was used to detect the presence of non-neutralizing antibody against DENV-1 (WP) virus.

### Data analysis

The present study is largely descriptive. Comparisons of the incidence of solicited adverse events between groups, neutrophil count parameters (baseline, absolute and relative decline, day of nadir) between groups, and comparisons of the ages of vaccinees and placebo recipients were performed using a 2-tailed Fisher Exact Test. Comparisons of mean peak titer, onset of viremia, duration of viremia, were performed using Tukey-Kramer HSD test. Mean values ± standard error (SE) are indicated. Statistical analysis was performed using JMP software (version 5.0.1.2; SAS Institute).

## Results

### Enrollment

**Figure 1:**
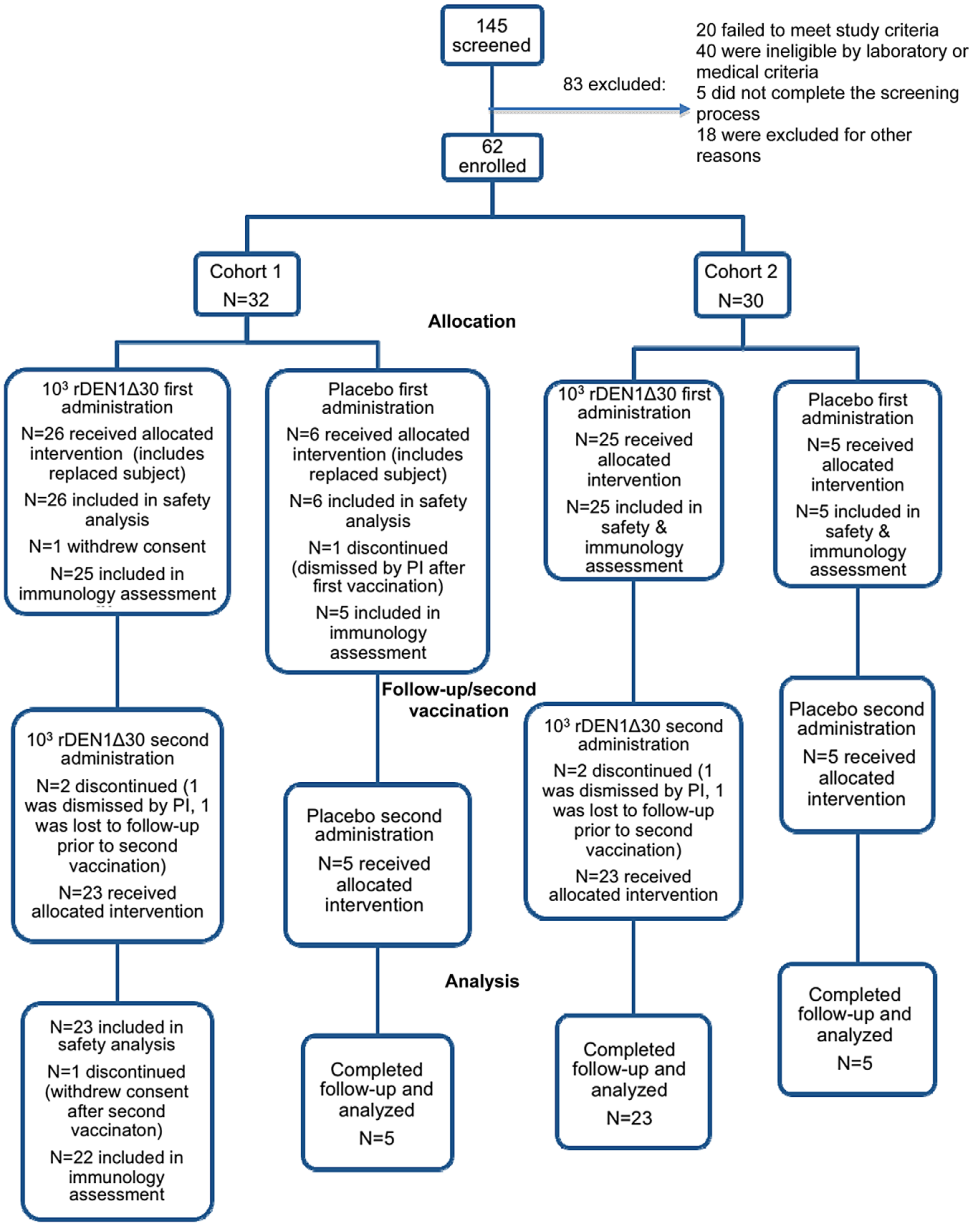
Screening, enrollment, vaccination, and follow-up summary of subjects enrolled in the trial evaluating rDEN1Δ30.

A total of 145 subjects were screened and 62 subjects were enrolled in the trial (**Figure 1**). All subjects who received vaccine (or placebo) were included in the safety assessment even if they did not complete day 42 and were replaced. Two subjects were replaced; one had received vaccine and the other had received placebo. Fifty-one subjects received the first dose of vaccine (26 in cohort 1 and 25 in cohort 2) and 11 subjects received the first dose of placebo (6 in cohort 1 and 5 in cohort 2). Forty-six subjects (23 in each cohort) received a second vaccination and 10 subjects received placebo (5 in each cohort) at second vaccination; all were included in the safety assessment. One vaccinee in cohort 1 withdrew prior to study day 222 and was not included in the immunological assessment (Figure 1). A statistically significant difference in age was neither observed between vaccinees (32±1.4 years) and placebo recipients (36±2.5 years), nor between the two cohorts (data not shown). Twenty-six subjects (42%) were female and 36 subjects (58%) were male. This difference was not significant. Thirty-one of 51 vaccinees (61%) self-reported as Black, 17 (33%) as White, and the remaining 6% were comprised of multi-racial, Pacific Islander, or unknown racial identity. In comparison, 9/11 placebo recipients (82%) self-reported as Black and the remaining 18% self-reported as White.

### Adverse events following Dose 1

The type and frequency of adverse events reported by subjects who received this new lot of rDEN1Δ30 (Lot DEN1#104A) were compared with those reported by subjects who received the Lot DEN1#1 to establish bioequivalence and to expand our safety database. Two SAEs occurred following dose 1 in a single vaccine-recipient. This subject fractured his ankle and, while hospitalized for surgical repair of the ankle fracture (SAE #1), had surgical repair of a pre-existing spinal stenosis (SAE #2). Both SAEs were judged to be unrelated to vaccination. The vaccine was well tolerated by all vaccinated subjects. None of the subjects developed a dengue-like illness. One vaccinee developed mild injection site erythema starting 4 days post-vaccination and lasting for 4 days. None of the other subjects developed injection site erythema, induration, or tenderness during the intensive follow-up period (16 days post-vaccination). The frequency and severity of solicited reactogenicity events following vaccination with rDEN1Δ30, Lot DEN1#104A, was comparable to that reported following vaccination with the previous lot, DEN1#1[22], and selected reactogenicity events are shown in **Table 1**.

**Table 1:** Clinical and virology data from subjects vaccinated with rDEN1Δ30 or placebo.

					No. subjects with indicated clinical sign/symptom (%)				
Treatment	Cohort and dosing schedule	Dose #	N	No. viremic (%)	Fever	Rash	Headache	Neutropenia^1^	↑ALT^2^
DEN1Δ30 (DEN1#1)^3^	Single dose	1	20	9 (43)	1^4^ (5)	9 (40)	8 (40)	9^5^ (45)	0 (0)
DEN1Δ30 (DEN1#104A)	Cohort 1, 0+120	1	26	17 (65)	0 (0)	8 (31)	11 (42)	11 (42)	1 (4)
DEN1Δ30 (DEN1#104A)	Cohort 2, 0+180	1	25	17 (68)	0 (0)	6 (24)	10 (40)	12 (48)	0 (0)
Placebo^6^	Placebo	1	11	0 (0)	0 (0)	0 (0)	2 (18)	2 (18)	0 (0)
DEN1Δ30 (DEN1#104A)	Cohort 1, 0+120	2	23	0 (0)	0 (0)	0 (0)	10 (43)	1 (4)	1 (4)
DEN1Δ30 (DEN1#104A)	Cohort 2, 0+180	2	23	0 (0)	0 (0)	0 (0)	7 (30)	3 (13)	1 (4)
Placebo^7^	Placebo	2	10	0 (0)	0 (0)	0 (0)	1(10)	1(10)	0 (0)

1 Neutropenia is defined as an ANC ≤1500/µL.

2 The upper limit of normal (ULN) for ALT is 54/µL (63/µL) for females (males). ALT>1.25×ULN is recorded as elevated.

3 Historical data [28].

4 Fever was due to unrelated viral pharyngitis.

5 In this previously reported study neutropenia was defined as <1500/µL. Eight subjects were reported to be neutropenic. One additional subject had an ANC of 1500/µL. This subject is included in the current analysis.

6 Placebo recipients from only the first dose of the 2-dose study are presented.

7 Placebo recipients from only the second dose of the 2-dose study are presented.

Overall, 42/51 subjects (82%) who received rDEN1Δ30, Lot DEN1#104A developed at least one solicited clinical or laboratory adverse event, compared with 5/11 placebo recipients (45%), p = 0.02. The most commonly observed adverse events were rash, headache, and neutropenia. Since the frequencies of adverse events were similar for cohort 1 and cohort 2 (shown separately in **Table 1**), the pooled data is discussed here. The only solicited adverse event that showed a trend towards more common occurrence in vaccine recipients was rash (27% of vaccinees, 0% placebo recipients, p = 0.06). A mild asymptomatic maculopapular rash, similar in character to what we have described previously, was detected in 14 vaccinees (**Table 1**) [22], [23], [24]. The mean onset of rash was study day 12.8±0.4 and the mean duration was 11±2.2 days. Because the rash was not noticed by the majority of the volunteers, the reported duration of rash may be longer than its actual duration because subjects were not seen between study days 21 and 28 and the rash was not determined to be resolved until the subject was evaluated by a clinician at study day 28. There were 39 episodes of headache reported by 21 vaccinees throughout the 42-day post-vaccination period. The mean duration of headache was 2.2±0.6 days with a mean day of onset of 10.1±1.2. Sixty-nine percent of headache episodes were of mild severity and 31% were of moderate severity. There were four episodes of headache reported by 2 placebo recipients; this difference was not statistically significant when compared with vaccinees. Seven vaccinees (14%) reported mild (6 subjects) or moderate (1 subject) retro-orbital pain following dose 1, six of them between day 13 and 16. None of the subjects developed fever during the 16-day follow-up. Four vaccinees reported myalgia that was determined to be possibly, probably, or definitely related to vaccine. Three vaccinees (6%) reported a mild myalgia lasting 3–5 days; one vaccinee reported moderate myalgia lasting 2 days. One placebo recipient (9%) reported myalgia lasting 3 days.

Twenty-three of 51 vaccinees (45%) in the current study (Lot DEN1#104A) developed transient neutropenia, similar to the frequency observed in our previous study (**Table 1**). Neutropenia in vaccinees was graded as mild (range 1,000–1,500/mm^3^) in 15 subjects, moderate (range 750–999/mm^3^) in 3 subjects , and severe (range 500–749/mm^3^) in 5 subjects. The onset of neutropenia ranged from Study Day 4 to Study Day 16, [mean day of onset  = 11.8±0.7 (SE)] and resolved in all subjects. The mean duration of neutropenia in vaccinees was 2.4 days ±0.3 days. Four of 5 subjects with severe neutropenia had an ANC<750/mm^3^ on a single day and one subject had an ANC<750/mm^3^ on days 12 and 14. Two of 11 placebo recipients (18%) developed neutropenia, both episodes were mild. In the current study, vaccinees who became neutropenic had a statistically significant lower mean baseline ANC (2,663/mm^3^) than vaccinees who did not become neutropenic (3,855/mm^3^), α = 0.01 (**Table 2**). Individuals who had a baseline ANC of ≤3,000/mm^3^ were more likely to become neutropenic than individuals with baseline ANCs > 3,000/mm^3^ (p<0.0001). Sixteen of the 23 vaccinees (70%) who became neutropenic had a baseline ANC of ≤3,000/mm^3^ compared with 4/28 (14%) vaccinees who did not become neutropenic. The mean absolute decline in ANC was not significantly different between those vaccinees who became neutropenic and those who remained non-neutropenic. However, the mean decline in ANC among all vaccinees was significantly greater than in placebo recipients (**Table 2**). One vaccinee developed a moderate elevation in serum ALT activity that was possibly related to vaccine. The ALT level peaked on day 15 at 123 IU/L and was not associated with nausea, vomiting, abdominal pain, or hepatomegaly. Although at screening this subject denied taking any prescription drugs, she later indicated that she had been taking anti-depressive medication for several weeks, which may have contributed to the elevation in serum ALT level.

**Table 2:** Absolute neutrophil counts (ANC) in neutropenic rDEN1Δ30 vaccinees, non-neutropenic rDEN1Δ30 vaccinees, and placebo recipients following a single dose of vaccine.

Group	No. subjects	Baseline ANC[Stat group]^1^	Mean absolute decline in neutrophils[Stat group]^2^	Mean % decline[Stat group]^2^	Mean day of ANC nadir[Stat group]^2^
Vaccine (neutropenic)	23	2,663 [A]	1,594 [A]	56% [A]	14.3±0.8 [A]
Vaccine (non-neutropenic)	28	3,855 [B]	1,440 [A]	36% [B]	12.0±0.8 [AB]
Placebo	10	3,100 [AB]	501[B]	17% [C]	9.0±1.3 [B]

1 Means not assigned the same letter are significantly different (α = 0.01).

2 Means not assigned the same letter are significantly different (α = 0.05).

### Adverse events following Dose 2

Following Dose 2, none of the subjects developed dengue-like illness, fever, retro-orbital pain, or rash (**Table 1**). A statistically significant difference in the number of volunteers reporting headache between vaccinees and placebo recipients was not observed. The incidence of neutropenia following second vaccination was similar in vaccine and placebo recipients; four vaccine recipients (9%) developed mild neutropenia and one placebo recipient (10%) developed moderate neutropenia. Two vaccinees developed a mild transient elevation in serum ALT level (peak of 82 IU/L on day 4 and peak of 83 IU/L on day 7 post dose 2, respectively). The elevated ALT level in both subjects resolved within 4 days. Neither of these subjects met the definition of infection following second dose nor did they have any increase in ELISA antibody titer following the second dose.

### Assessment of viremia and DENV-1 antibody response

Thirty-four of 51 vaccinees (67%) who received rDEN1Δ30 Lot DEN1#104A had detectable viremia following the first vaccination (**Table 3**). The percent of volunteers with viremia, the mean peak virus titer, and the mean number of viremic days following dose 1 in the current study were comparable to that observed in the previous study rDEN1Δ30 Lot DEN1#1 [22]. Following dose 1, 94% vaccinees were infected with rDEN1Δ30 Lot DEN1#104A (88% in cohort 1 and 100% in cohort 2) and 95% with rDEN1Δ30 LotDEN1#1. Amongst the 48 subjects infected with rDEN1Δ30 Lot DEN1#104A, one was viremic on day 12 but did not meet criteria for seroconversion, resulting in an overall seroconversion rate of 92% to wild-type DENV-1 virus by study day 42 (84% in cohort 1 and 100% in cohort 2, **Table 4**). With regard to immunogenicity, PRNT_60_ titers against wild-type DENV-1 induced by the two lots of vaccine were comparable (less than a 2-fold difference) (**Table 4**).

**Table 3:** Viremia onset, duration, and mean peak titer following a first dose of rDEN1Δ30.

rDEN1Δ30 Lot #	Cohort and dosing schedule	Dose	N	No (%) infected^1^	No (%) with viremia	Mean peak titer ±SE (log_10_ PFU/mL)^2^	Mean day of onset of viremia ±SE	Mean # of days of viremia ±SE
DEN1#1^3^	Single dose	1	20	19 (95)	9 (45)	1.0±0.2	9.8±0.7	2.8±0.8
DEN1#104A	Cohort 1, 0+120	1	26	23 (88)	17 (65)	1.1±0.1	10.1±0.5	3.4±0.5
DEN1#104A	Cohort 2, 0+180	1	25	25 (100)	17 (68)	1.0±0.2	10.8±0.7	3.6±0.5
Aggregate data for all subjects:			71	67 (94)	43 (61)	1.0±0.1	10.3±0.4	3.3±0.3

1 Infected is defined as recovery of vaccine virus from the blood and/or a ≥4-fold rise in PRNT_60_ by day 42 post vaccination compared with day 0.

2 Mean peak titer is calculated for viremic subjects only.

3 Historical Data [28].

**Table 4:** Serum neutralizing antibody response induced by a single 10^3^ PFU dose of rDEN1Δ30.

rDEN1Δ30 Lot #	Cohort and dosing schedule	N	% infected^2^ (%)	Reciprocal geometric mean PRNT_60_ (range)^1^		% seroconversion by PRNT^3^
				Day 0	Day 42	
DEN1#1^4^	Single dose	20	95	<5	181 (8–1242)	95
DEN1#104A	Cohort 1, 0+120	25	88	<5	106 (<5–527)	84
DEN1#104A	Cohort 2, 0+180	25	100	<5	169 (7–965)	100
Aggregate data		70	94	-	142 (<5–1242)	93

1 Plaque reduction neutralization titer 60%. Geometric mean titers calculated for all subjects who developed detectable antibody, including those who did not seroconvert (see footnote 3).

2 Infection is defined as recovery of vaccine virus from the blood and/or seroconversion to DEN1.

3 Seroconversion is defined as a ≥4-fold rise in PRNT_60_ at day 28 or 42 post-vaccination.

4 Previous trial. All serum samples from the current and previous trial were re-assayed concurrently.

Twenty-three vaccinees per cohort received a second dose of vaccine (**Figure 1**). Following a second dose of vaccine at either interval, viremia was not detected in any subject (**Table 1**), and none of the subjects had a 4-fold or greater rise in serum neutralizing antibody titer against DENV-1, indicating that none of the subjects met the protocol definition of infection following a second dose of rDEN1?30 (**Table 5**). Geometric mean PRNT_60_ titers against wild-type DENV-1 following the second dose of vaccine were similar in cohorts 1 and 2 (**Table 5**). Three of the four subjects who did not seroconvert after the first dose of vaccine received a second dose of vaccine four months later; none of them developed a ≥4-fold rise in serum neutralizing antibody titer or in ELISA antibody titer. Of the subjects who seroconverted to DENV-1 after the first vaccination, one who received a second dose of vaccine four months after the first dose developed a ≥4-fold rise in ELISA antibody titer following the second dose, as did four subjects who received a second dose six months after the first dose (**Table 5**). These findings suggest that a single dose of rDEN1Δ30 was highly infectious and was able to provide sterilizing humoral immunity to infection with the vaccine virus for at least 6 months in 83% of vaccinees.

**Table 5:** Serum antibody response induced by a second 10^3^ PFU dose of rDEN1Δ30 given at 4 or 6 months after first dose.

rDEN1Δ30 Lot#	Cohort and dosing schedule	N	% infected^1^	Reciprocal geometric mean PRNT_60_ (range)^1^			% Boosted by PRNT^5^	No (%) with ≥4-fold rise in ELISA titer
				Day 0^2^	Day 28^3^	Day 42^4^		
DEN1#104A	Cohort 1,(0+120)	22	0	39 (<5–300)	38 (<5–284)	36 (<5–176)	0^5^	1 (5)
DEN1#104A	Cohort 2, (0+180)	23	0	37 (<5–361)	36 (5–284)	31 (<5–180)	0^5^	4 (17)

1 Infection is defined as recovery of vaccine virus from the blood and/or seroconversion to DEN1 by PRNT_60_.

2 Day 0 is day of second vaccination (day 120 for 0/120 cohort and day 180 for 0/180 cohort).

3 Day 28 is day 28 post-second vaccination.

4 Day 42 is day 42 post-second vaccination

5 Boost is defined as a ≥4-fold rise in PRNT_60_ at day 28 or 42 post-vaccination.

## Discussion

The development of a safe and effective tetravalent dengue vaccine has been a goal for decades, yet a licensed vaccine is still not available. At a minimum, such a vaccine must have an acceptable safety profile and must induce long-lived protective immunity against all four serotypes of wild type dengue virus. Ideally this would be accomplished with a single dose of vaccine. However, tetravalent vaccines currently in clinical development require two or three doses [14], [18]. A number of dengue viruses have been attenuated either by serial passage in tissue culture or by the introduction of attenuating mutations and/or chimerization of dengue viruses using recombinant DNA technology [26], [29], [30], [31], [32]. In addition, a tetravalent vaccine based on chimerization of DENV-1-4 with the yellow fever vaccine virus is currently in Phase II/III clinical development [14], [20]. Other investigational vaccines have been abandoned because they were either under or over-attenuated [15], [16], [17], [33], [34]. In addition, there are concerns that viral interference may affect the ability of a live attenuated tetravalent dengue vaccine to induce a balanced immune response to all four serotypes. For these reasons, we have attempted to carefully examine the safety profile and humoral immune response to each monovalent DENV vaccine candidate virus prior to formulating a tetravalent vaccine. Clinical examination and laboratory studies performed every other day for the first 16 days post vaccination have helped to fully characterize the reactogenicity and kinetics of replication of these viruses when given as monovalent vaccines.

The ability of live attenuated dengue vaccines to induce a satisfactory antibody response without clinically significant reactogenicity has been a hurdle to DENV vaccine development. There are few published studies of monovalent DENV-1 vaccines to which we can compare the safety and immunogenicity of rDEN1Δ30 in healthy adult subjects. Three such studies describe the DENV-1 vaccine candidates 16007, 45AZ5 PDK20, and 45AZ5 PDK27 [17], [35], [36]. These candidates were attenuated by serial passage in tissue culture, and the parent virus of 45AZ5 PDK20 and 45AZ5 PDK27 underwent chemical mutagenesis prior to passage in tissue culture. Five subjects received DENV-1 16007 as a monovalent vaccine and 12 subjects received 45AZ5 PDK20. Vaccine candidate 16007 was well tolerated: none of the subjects developed an oral temperature >38°C, one subject developed a rash and 2 subjects developed elevated liver function tests [35]. However, only 60% of vaccinees seroconverted to the vaccine as defined as a PRNT_50_ ≥1∶10.In contrast, 100% of vaccinees seroconverted to DEN1 after receipt of the monovalent DENV-1 vaccine 45AZ5 PDK20; however, 5/12 subjects developed a temperature >38°C and 3/12 developed dengue-like illness [17]. Additionally, 20% to 35% of subjects who received a tetravalent formulation containing 45AZ5 PDK20 developed a temperature of >38°C [18]. The further passaged DENV-1 candidate vaccine 45AZ5 PDK27 was evaluated as a monovalent DENV-1 vaccine in 10 subjects and was found to be more attenuated than 45AZ5 PDK20 [36]. While the reactogenicity profile of 45AZ5 PDK27 was acceptable, only 40% of vaccinees seroconverted to DENV-1 following a single dose of 45AZ5 PDK27 [36]. Although 45AZ5 PDK27 was less immunogenic than 45AZ5 PDK20, it has replaced 45AZ5 PDK27 in the tetravalent formulations currently under evaluation, illustrating the trade-off that is sometimes necessary to balance reactogenicity and immunogenicity. Thus, achieving a satisfactory balance between attenuation and immunogenicity has been difficult to attain by passage and/or mutagenesis of DENV-1. In the rDEN1Δ30 vaccine virus, the attenuating 30 nucleotide deletion is located in the 3′ UTR but the virus is otherwise wild-type. This might have contributed to its high level of infectivity and immunogenicity.

The promising safety profile of the rDEN1Δ30 vaccine described in a previous single-dose study was affirmed in the current two-dose study [22]. None of the subjects developed fever, a dengue-like illness or any study-related SAE. All of the clinical solicited adverse events were mild or moderate in severity and were transient in nature. As we have reported for our previous dengue vaccine studies, neutropenia and rash were the most commonly reported adverse events in subjects who received rDEN1Δ30. As was seen in the previous study of this vaccine candidate, the onset of neutropenia and rash generally followed the onset of viremia. The definition of neutropenia used in our studies is more inclusive than that utilized in many other dengue vaccine studies. We defined neutropenia as an ANC of ≤1500/mm^3^ and obtained blood counts every other day for the first 16 days after vaccination; other studies of leading dengue vaccine candidates have defined neutropenia as an ANC<1,000/mm^3^ with less frequent monitoring, e.g. only on day 15, or on days 4, 8, 12, and 16 post vaccination [14], [17], [18], [20]. Had we used an ANC<1,000/mm^3^ to define neutropenia our studies, only 2/71 (ANC measured on day 16 only) or 5/71 (ANC measured on days 4, 8, 12, and 16 only) vaccine recipients would have been described as neutropenic, a rate of neutropenia comparable to that reported in the studies indicated above. In addition, more than 60% of our study subjects were of African descent, a population known to maintain a lower mean baseline ANC [37], [38], [39]. In comparison, subjects of African descent made up only 5–33% of volunteers in the studies referenced above. Most importantly, the very short duration of neutropenia observed in recipients of rDEN1Δ30 was not associated with fever or other clinical signs or symptoms suggestive of neutropenia-related illness. In contrast to neutropenia associated with neoplasia or therapeutic interventions, we assume that neutrophil function during DENV infection or following vaccination with a live-attenuated DENV vaccine is not compromised and that the short duration of neutropenia does not compromise protective immune functions. Our monovalent DENV vaccines have been evaluated in approximately 500 subjects thus far and fever of unknown origin or any sign or symptom suggestive of opportunistic infections has not been observed. To our knowledge, neutropenia due to natural dengue illness has not been associated with secondary bacteremia. A review of cases of concomitant bacteremia in patients diagnosed with DHF found that leukopenia was not associated with an increased incidence of bacteremia compared with control patients [40].

The investigational vaccine rDEN1Δ30 appears to have a well-balanced safety and immunogenicity profile. In addition to the acceptable reactogenicity of rDEN1Δ30 described above, the vaccine was able to induce an overall seroconversion rate of 93% (65/70 subjects) when administered as a single subcutaneous dose to flavivirus-naïve healthy adults. Importantly, the immunogenicity end-point used in both this and our previously reported study of rDEN1Δ30 was a 4-fold rise in serum neutralizing antibody (i.e., a PRNT_60_ titer of ≥1∶20 defined seroconversion) and is more stringent compared with a PRNT_50_ titer of ≥1∶10 used by others in the studies described above. In addition to the high seroconversion rate that was achieved following a single dose of vaccine, rDEN1Δ30 induced sterilizing humoral immunity to infection with a second dose of vaccine administered 4 or 6 months later in the vast majority of vaccinees, a finding that could be indicative of long-term homotypic protection as has been described following natural DENV infection [10], [41].

In the present study, the four subjects who did not develop detectable neutralizing antibody after the first dose of rDEN1Δ30 could not be infected by a second dose of vaccine given 4 months later. Following dose 1, one of the 4 subjects had laboratory findings consistent with infection by the vaccine virus, and another subject had findings that may be suggestive of infection by the vaccine virus. The former had detectable viremia on study day 12 and a mild neutropenia on study day 16, and the latter subject was mildly neutropenic on study day 4 following first vaccination. The other two subjects did not have clinical or laboratory findings suggestive of infection. Of these four subjects, three received a second dose of vaccine but did not develop a detectable neutralizing antibody response following dose 2. It is unclear whether these individuals were vaccine non-responders, completely resistant to infection, or whether their innate immune system was able to abort infection prior to the induction of an antibody response. Loss of vaccine potency was excluded as a cause for the absence of infection following dose 2.

This study has important implications for the development of a live attenuated tetravalent dengue vaccine. First, the expanded safety evaluation of the monovalent rDEN1Δ30 vaccine confirmed it to be well tolerated without vaccine-related fever or dengue-like illness. Second, the vaccine was able to induce seroconversion to DENV-1 in 93% of recipients when given as a single subcutaneous dose of 10^3^ PFU. The 10^3^ PFU dose required for the induction of seroconversion to DENV-1 and the ability to grow this virus to high titer (>10^7^ PFU/mL), make this a very economical vaccine component to produce. Third, the vaccine virus behaved immunologically much like wild-type virus in that sterilizing homotypic immunity was induced after a single dose. It is unknown whether this will make boosting of the humoral response to DENV-1 more difficult should multiple doses of the tetravalent vaccine be required. It is also not known whether the observed protection against a second dose of vaccine virus will translate into protection against infection/disease following exposure to wild type DENV-1 virus after vaccination. Future studies with tetravalent vaccine will need to evaluate whether boosting is necessary and will need to be designed to determine a suitable interval for boosting. Lastly, because the replication kinetics of this virus have been very well characterized as a monovalent vaccine, any effect of viral interference on the replication kinetics of the vaccine when administered as part of a tetravalent formulation should be discernable. In summary, rDEN1Δ30 is an excellent candidate for inclusion in live tetravalent dengue vaccine formulations, and clinical evaluation of tetravalent dengue vaccines with this rDEN1Δ30 as DENV-1 component have been initiated.

Our study has at least two potential limitations regarding the infectivity, safety, and immunogenicity of rDEN1Δ30 when administered as a component of a tetravalent formulation. First, sterilizing humoral immunity to a second dose of monovalent rDEN1Δ30 vaccine at 6 months might not translate into sterilizing humoral immunity six months after vaccination with a tetravalent vaccine. It is not known whether or not the high seroconversion rates achieved following a single dose of the rDEN1Δ30 vaccine, when given as a monovalent virus, will be achievable when the candidate vaccine is included in a tetravalent formulation. Secondly, the results of our studies in flavivirus-naive healthy adults do not necessarily mirror how this investigational vaccine will behave in flavivirus-naive and partially immune children, one of the target groups for vaccination in several hyperendemic areas. Tetravalent vaccine studies will need to carefully proceed from adults into children and from flavivirus-naive into partially immune individuals to evaluate the safety and immunogenicity of this vaccine in these vulnerable populations.

## Supporting Information

Checklist S1 Consort checklist.
(DOC)

Protocol S1 (PDF)

Text S1 IRB approvals.
(PDF)

Text S2 NIH cover sheet.
(PDF)

## References

[1] Halstead SB, Suaya JA, Shepard DS (2007). The burden of dengue infection.. Lancet.

[2] Mammen MP, Pimgate C, Koenraadt CJ, Rothman AL, Aldstadt J (2008). Spatial and temporal clustering of dengue virus transmission in Thai villages.. PLoS Med.

[3] Honorio NA, Nogueira RM, Codeco CT, Carvalho MS, Cruz OG (2009). Spatial evaluation and modeling of Dengue seroprevalence and vector density in Rio de Janeiro, Brazil.. PLoS Negl Trop Dis.

[4] WHO (2009). Dengue Guidelines for diagnosis, treatment, prevention, and control..

[5] Kyle JL, Harris E (2008). Global spread and persistence of dengue.. Annu Rev Microbiol.

[6] Simmons CP, Farrar J (2009). Changing patterns of dengue epidemiology and implications for clinical management and vaccines.. PLoS Med.

[7] Nisalak A, Endy TP, Nimmannitya S, Kalayanarooj S, Thisayakorn U (2003). Serotype-specific dengue virus circulation and dengue disease in Bangkok, Thailand from 1973 to 1999.. Am J Trop Med Hyg.

[8] Vaughn DW, Green S, Kalayanarooj S, Innis BL, Nimmannitya S (2000). Dengue Viremia Titer, Antibody Response Pattern, and Virus Serotype Correlate with Disease Severity.. J Infect Dis.

[9] Imrie A, Meeks J, Gurary A, Sukhbaatar M, Truong TT (2007). Antibody to dengue 1 detected more than 60 years after infection.. Viral Immunol.

[10] Sabin A (1952). Research on dengue during World War II.. Am J Trop Med Hyg.

[11] Papaevangelou G, Halstead SB (1977). Infections with two dengue viruses in Greece in the 20th century. Did dengue hemorrhagic fever occur in the 1928 epidemic?. Am J Trop Med Hyg.

[12] Guzman MG, Kouri GP, Bravo J, Soler M, Vazquez S (1990). Dengue hemorrhagic fever in Cuba, 1981: a retrospective seroepidemiologic study.. Am J Trop Med Hyg.

[13] Burke DS, Nisalak A, Johnson DE, Scott RM (1988). A prospective study of dengue infections in Bangkok.. Am J Trop Med Hyg.

[14] Morrison D, Legg TJ, Billings CW, Forrat R, Yoksan S (2010). A novel tetravalent dengue vaccine is well tolerated and immunogenic against all 4 serotypes in flavivirus-naive adults.. J Infect Dis.

[15] Sabchareon A, Lang J, Chanthavanich P, Yoksan S, Forrat R (2002). Safety and immunogenicity of tetravalent live-attenuated dengue vaccines in Thai adult volunteers: role of serotype concentration, ratio, and multiple doses.. Am J Trop Med Hyg.

[16] Sabchareon A, Lang J, Chanthavanich P, Yoksan S, Forrat R (2004). Safety and immunogenicity of a three dose regimen of two tetravalent live-attenuated dengue vaccines in five- to twelve-year-old Thai children.. Pediatr Infect Dis J.

[17] Sun W, Edelman R, Kanesa-Thasan N, Eckels KH, Putnak JR (2003). Vaccination of human volunteers with monovalent and tetravalent live-attenuated dengue vaccine candidates.. Am J Trop Med Hyg.

[18] Sun W, Cunningham D, Wasserman SS, Perry J, Putnak JR (2009). Phase 2 clinical trial of three formulations of tetravalent live-attenuated dengue vaccine in flavivirus-naive adults.. Hum Vaccin.

[19] Simasathien S, Thomas SJ, Watanaveeradej V, Nisalak A, Barberousse C (2008). Safety and immunogenicity of a tetravalent live-attenuated dengue vaccine in flavivirus naive children.. Am J Trop Med Hyg.

[20] Lang J (2009). Recent progress on sanofi pasteur's dengue vaccine candidate.. J Clin Virol.

[21] Durbin AP, Karron RA, Sun W, Vaughn DW, Reynolds MJ (2001). Attenuation and immunogenicity in humans of a live dengue virus type-4 vaccine candidate with a 30 nucleotide deletion in its 3′-untranslated region.. Am J Trop Med Hyg.

[22] Durbin AP, McArthur J, Marron JA, Blaney JE, Thumar B (2006). The live attenuated dengue serotype 1 vaccine rDEN1Delta30 is safe and highly immunogenic in healthy adult volunteers.. Hum Vaccin.

[23] Durbin AP, Whitehead SS, McArthur J, Perreault JR, Blaney JE (2005). rDEN4 Delta 30, a Live Attenuated Dengue Virus Type 4 Vaccine Candidate, Is Safe, Immunogenic, and Highly Infectious in Healthy Adult Volunteers.. J Infect Dis.

[24] Durbin AP, McArthur JH, Marron JA, Blaney JE, Thumar B (2006). rDEN2/4Delta30(ME), A Live Attenuated Chimeric Dengue Serotype 2 Vaccine Is Safe and Highly Immunogenic in Healthy Dengue-Naive Adults.. Hum Vaccin.

[25] Blaney JE, Sathe NS, Goddard L, Hanson CT, Romero TA (2008). Dengue virus type 3 vaccine candidates generated by introduction of deletions in the 3′ untranslated region (3′-UTR) or by exchange of the DENV-3 3′-UTR with that of DENV-4.. Vaccine.

[26] Whitehead SS, Blaney JE, Durbin AP, Murphy BR (2007). Prospects for a dengue virus vaccine.. Nat Rev Microbiol.

[27] Blaney JE, Matro JM, Murphy BR, Whitehead SS (2005). Recombinant, Live-Attenuated Tetravalent Dengue Virus Vaccine Formulations Induce a Balanced, Broad, and Protective Neutralizing Antibody Response against Each of the Four Serotypes in Rhesus Monkeys.. J Virol.

[28] Whitehead SS, Falgout B, Hanley KA, Blaney JE, Markoff L (2003). A Live, Attenuated Dengue Virus Type 1 Vaccine Candidate with a 30-Nucleotide Deletion in the 3′ Untranslated Region Is Highly Attenuated and Immunogenic in Monkeys.. J Virol.

[29] Guirakhoo F, Kitchener S, Morrison D, Forrat R, McCarthy K (2006). Live attenuated chimeric yellow fever dengue type 2 (ChimeriVax-DEN2) vaccine: Phase I clinical trial for safety and immunogenicity: effect of yellow fever pre-immunity in induction of cross neutralizing antibody responses to all 4 dengue serotypes.. Hum Vaccin.

[30] Guirakhoo F, Pugachev K, Zhang Z, Myers G, Levenbook I (2004). Safety and efficacy of chimeric yellow Fever-dengue virus tetravalent vaccine formulations in nonhuman primates.. J Virol.

[31] Durbin AP, Whitehead SS (2010). Dengue vaccine candidates in development.. Curr Top Microbiol Immunol.

[32] Guy B, Saville M, Lang J (2010). Development of Sanofi Pasteur tetravalent dengue vaccine.. Hum Vaccin.

[33] Edelman R, Wasserman SS, Bodison SA, Putnak RJ, Eckels KH (2003). Phase I trial of 16 formulations of a tetravalent live-attenuated dengue vaccine.. Am J Trop Med Hyg.

[34] Kitchener S, Nissen M, Nasveld P, Forrat R, Yoksan S (2006). Immunogenicity and safety of two live-attenuated tetravalent dengue vaccine formulations in healthy Australian adults.. Vaccine.

[35] Kanesa-thasan N, Sun W, Kim-Ahn G, Van Albert S, Putnak JR (2001). Safety and immunogenicity of attenuated dengue virus vaccines (Aventis Pasteur) in human volunteers.. Vaccine.

[36] Edelman R, Tacket CO, Wasserman SS, Vaughn DW, Eckels KH (1994). A live attenuated dengue-1 vaccine candidate (45AZ5) passaged in primary dog kidney cell culture is attenuated and immunogenic for humans.. J Infect Dis.

[37] Haddy TB, Rana SR, Castro O (1999). Benign ethnic neutropenia: what is a normal absolute neutrophil count?. J Lab Clin Med.

[38] Hsieh MM, Everhart JE, Byrd-Holt DD, Tisdale JF, Rodgers GP (2007). Prevalence of neutropenia in the U.S. population: age, sex, smoking status, and ethnic differences.. Ann Intern Med.

[39] Reed WW, Diehl LF (1991). Leukopenia, neutropenia, and reduced hemoglobin levels in healthy American blacks.. Arch Intern Med.

[40] Lee IK, Liu JW, Yang KD (2005). Clinical characteristics and risk factors for concurrent bacteremia in adults with dengue hemorrhagic Fever.. Am J Trop Med Hyg.

[41] Halstead SB, Casals J, Shotwell H, Palumbo N (1973). Studies on the immunization of monkeys against dengue. I. Protection derived from single and sequential virus infections.. Am J Trop Med Hyg.

