# Supplementary material for: A Single Dose of the DENV-1 Candidate Vaccine rDEN1Δ30 Is Strongly Immunogenic and Induces Resistance to a Second Dose in a Randomized Trial
Source: PLoS Negl Trop Dis. 2011 Aug 2;5(8):e1267. doi: 10.1371/journal.pntd.0001267 (PMC3149013; doi:10.1371/journal.pntd.0001267)
Supplement: Protocol S1 — (PDF) [file pntd.0001267.s002.pdf]

Safety and Immunogenicity of a 2-Dose Regimen of rDEN1Δ30 Dengue Serotype 1 Vaccine with Boosting at 4 versus 6 Months

CIR Protocol Number	229
WIRB Protocol Number	20070718
FDA IND Number	BB-IND 11677
Ionizing Radiation	None
Multi-institution	No
Project Assurance:	FWA #00000287

Protocol Version:	5.0
Date:	09 October 2008

Sponsored by:
Regulatory Compliance and Human Subjects Protection Branch (RCHSPB)
National Institute of Allergy and Infectious Diseases (NIAID)
National Institutes of Health (NIH)

The information contained herein is the property of the National Institute of Allergy and Infectious Diseases, National Institutes of Health and may not be reproduced, published, or disclosed to others without the written authorization of the National Institute of Allergy and Infectious Diseases.

Team Roster

**Principal Investigator:**	Anna Durbin, M.D.<br>Center for Immunization Research (CIR)<br>Johns Hopkins School of Public Health<br>624 N. Broadway<br>Baltimore, MD 21205<br>410-614-4736 (phone)
**Clinical Investigators:**	Priscilla Brooks, R.N., CCRC<br>Dan Elwood, R.N., CCRC<br>E. Wangeci Kagucia, MHS<br>Center for Immunization Research<br>Johns Hopkins School of Public Health<br>624 N. Broadway<br>Baltimore, MD 21205
**Scientific Investigators:**	Brian Murphy, M.D.<br>Stephen S. Whitehead, Ph.D.<br>Alexander C. Schmidt, M.D.<br>Laboratory of Infectious Diseases<br>NIAID, NIH<br>301-402-7754 (phone)
**RCHSPB Regulatory Affairs:**	John Tierney, R.N.<br>RCHSPB/NIAID/NIH<br>6700-B Rockledge Dr. Room #1112<br>MSC 7609<br>Bethesda, MD 20892<br>301-451-5136 (phone)
**Clinical Study Site:**	Center for Immunization Research<br>Johns Hopkins School of Public Health<br>624 N. Broadway<br>Baltimore, MD 21205<br>410-955-1622 (phone)<br><br>**and**<br><br>2112 F Street, Suite 305<br>Washington DC 20037<br>202-223-6564 (phone)

**Immunology Laboratories:**	Center for Immunization Research Laboratories<br>E5402 Hygiene Building<br>615 N. Wolfe Street<br>Baltimore, MD 21205<br>410-955-7230 (phone)
**Clinical Laboratories:**	University of Maryland Medical System<br>Laboratories of Pathology<br>22 South Green Street<br>Baltimore, MD 21201<br>410-328-1237 (phone)
	Quest Diagnostics<br>1901 Sulphur Springs Road<br>Baltimore, MD 21227<br>800-368-2576 (phone)
	Other CLIA certified laboratories may be used **only** if the primary clinical laboratories cannot provide the required tests in a timely manner.

Abbreviations

AE	adverse event
ALT	alanine transaminase
ANC	absolute neutrophil count
CBC	complete blood count
cGMP	current Good Manufacturing Practice
CIR	Center for Immunization Research
CLIA	Clinical Laboratory Improvement Amendments
CPK	creatine phosphokinase
CRF	case report form
DEN	dengue virus (serotypes DEN1, DEN2, DEN3, and DEN4)
DHF	dengue hemorrhagic fever
DSMB	Data and Safety Monitoring Board
DSS	dengue shock syndrome
ELISA	enzyme-linked immunosorbent assay
FDA	Food and Drug Administration
GCP	Good Clinical Practice
HBsAg	hepatitis B surface antigen
HCG	human choriogonadotropin
HCV	hepatitis C virus
HIV	human immunodeficiency virus
IND	investigational new drug
IRB	Institutional Review Board
LLN	lower limit of normal
NIAID	National Institute of Allergy and Infectious Diseases
NIH	National Institutes of Health
PBMC	peripheral blood mononuclear cell
PFU	plaque-forming units (virus titer)
PT	prothrombin time
PTT	partial thromboplastin time
RCHSPB	Regulatory Compliance and Human Subjects Protection Branch
SAE	serious adverse event
SAIC	Science Applications International Corporation
SCID	severe combined immunodeficiency disease
ULN	upper limit of normal
WHO	World Health Organization
WIRB	Western Institutional Review Board
WP	Western Pacific (dengue wild-type strain)

TABLE OF CONTENTS

Team Roster .... **ii**

Abbreviations .... **iv**

List of Tables .... **vii**

List of Figures .... **vii**

1.0 PROTOCOL SUMMARY **1**

2.0 INTRODUCTION **2**
- 2.1 Background – Dengue 2
- 2.2 Background – Human Experience with Dengue-1 3
- 2.3 Vaccine Description 3
  - 2.3.1 Final Container 4
  - 2.3.2 Composition 4
  - 2.3.3 Investigational Product Label (Enlarged Example) 4
- 2.4 Rationale 5
  - 2.4.1 Animal Experience 5
  - 2.4.2 Clinical Experience with rDEN1Δ30 5
- 2.5 Mosquito Transmissibility 9
- 2.6 Clinical Development Plan 9
- 2.7 Participation of Children 9
- 2.8 Statement of Compliance 9

3.0 OBJECTIVES **10**
- 3.1 Primary Objectives 10
- 3.2 Secondary Objectives 10

4.0 STUDY DESIGN **10**
- 4.1 Overall Design 10
- 4.2 Dosing Strategy 11
- 4.3 Sample Size and Estimated Duration of the Study 11
- 4.4 Treatment Assignment 12
- 4.5 Blinding 12
- 4.6 Duration of Participation 12

5.0 SELECTION AND ENROLLMENT OF VOLUNTEERS **13**
- 5.1 Inclusion Criteria 13
- 5.2 Exclusion Criteria 13
- 5.3 Access of Medical Records 14
- 5.4 Treatments that Could Potentially Interfere with Vaccine-Induced Immunity 14
- 5.5 Volunteer Withdrawal/Termination Criteria 15
- 5.6 Exclusion from Receipt of Second Vaccination 15
- 5.7 Replacement of Volunteers 16

6.0 VACCINE PREPARATION **17**
- 6.1 Supplies 17
- 6.2 Vaccine Storage 17
- 6.3 Vaccine Accountability 17

6.4 Disposition of Used/Unused Supplies 18

7.0 STUDY PROCEDURES 18
7.1 Recruitment and Screening 18
7.2 Screening Procedures (Up to 60 Days Prior to First Vaccination) 18
7.3 Immunization Procedure 19
7.4 Clinical Monitoring and Evaluation 19
7.5 Volunteer Temperature Diary 27
7.6 Clinical Laboratory Testing 27
7.7 Virologic Assays 27
7.8 Immunology Testing 28
7.8.1 Antibody Assay 28
7.8.2 Other Immunological Assays 28
7.9 Skin Biopsy 28

8.0 ADVERSE EVENT MONITORING 29
8.1 Definitions 29
8.1.1 Adverse Event 29
8.1.2 Serious Adverse Event 29
8.2 Assessment of Adverse Events 30
8.2.1 Identification of Adverse Events 30
8.2.2 Determination of Severity 30
8.2.3 Association with Receipt of Study Vaccine 32
8.3 Adverse Event Reporting 33
8.4 Adverse Event Monitoring 33
8.4.1 NIAID Intramural Data and Safety Monitoring Board 33
8.5 Stopping Criteria .... **Error! Bookmark not defined.**

9.0 DATA COLLECTION AND MONITORING 35
9.1 Source Documentation 35
9.2 Study Documentation 35
9.3 Retention of Specimens 35
9.4 Retention of Records 36
9.5 Protocol Revisions 36
9.6 Clinical Investigator’s Brochure 36
9.7 Study Monitoring 36

10.0 STATISTICAL CONSIDERATIONS 37
10.1 General Design 37
10.1.1 Description of the Statistical Methods to be Employed 37
10.1.2 Safety 38
10.1.3 Immunogenicity 38

11.0 PROTECTION OF HUMAN SUBJECTS 38
11.1 Institutional Review Board 38
11.2 Informed Consent 39
11.3 Risks 39
11.4 Venipuncture 39
11.5 Skin Biopsy 39

11.6 Immunization 39
11.7 Benefits 40
11.8 Compensation 40
11.9 Confidentiality 40
11.10 Biohazard Containment 40

12.0 PUBLICATION POLICY 41

13.0 REFERENCES 42

14.0 Appendix 1: Toxicity Table for Grading Adverse Events, Adapted from the HIV Vaccine Trials Network 44

List of Tables

Table 1: Immunization Schedule 1
Table 2: Magnitude, Onset, and Duration of Viremia in Volunteers Inoculated with 10^3 PFU of rDEN1Δ30, rDEN2/4Δ30, or rDEN4Δ30 7
Table 3: Serum Antibody Response Induced by 10^3 PFU of rDEN1Δ30, rDEN2/4Δ30, or rDEN4Δ30 7
Table 4: Clinical Summary of rDEN1Δ30, rDEN2/4Δ30(ME), and rDEN4Δ30 Candidate Vaccines 7
Table 5: AE Grading Table 30
Table 6: Assessment of Adverse Event Intensity 30
Table 7: Selected Laboratory Toxicity Table 32

List of Figures

Figure 1: Product Label for Lot DEN1#104A produced by Charles River Laboratories (example) 4
Figure 2: Solicited Adverse Events Recombinant Dengue Vaccines 8

1.0 PROTOCOL SUMMARY

Protocol Title: **Safety and Immunogenicity of a 2-Dose Regimen of rDEN1Δ30 Dengue Serotype 1 Vaccine with Boosting at 4 Versus 6 Months**

Draft Version: 5.0
Version Date: 09 October 2008

Revision History: **10 July 2007;** 05 June 2007**,** 18 May 2007**,** 08 March 2007

Volunteers: Healthy male and non-pregnant female volunteers 18 to 50 years of age with no history of previous flavivirus infection.

Number of Volunteers: 60 (2 groups of 30)

Study Design: Placebo-controlled, double-blind study evaluating the safety and immunogenicity of a live attenuated dengue 1 vaccine candidate boosted at 4 or 6 months.

Table 1: Immunization Schedule

Group	Total Volunteers	Dose
1	30<br>(25 Vaccinees, 5 Placebo recipients)	10^3 PFU vaccine (or placebo) given at Study Day 0 and 120
2	30<br>(25 Vaccinees, 5 Placebo recipients)	10^3 PFU vaccine (or placebo) given at Study Day 0 and 180

Product Description: The vaccine candidate rDEN1Δ30 is a live attenuated recombinant dengue virus type 1 (DEN1) that contains a 30 nucleotide deletion in the 3´ untranslated region of the genome.

2.0 INTRODUCTION

2.1 Background – Dengue

The World Health Organization (WHO) estimates that dengue viruses are responsible for more than 50 million cases of dengue fever and approximately one half million cases annually of the more severe disease, dengue hemorrhagic fever/shock syndrome (DHF/DSS) [1]. Infection with dengue viruses is the leading cause of hospitalization and death in children in at least eight tropical Asian countries [2]. There are four serotypes of dengue virus (DEN1, DEN2, DEN3, and DEN4) each capable of causing dengue illness ranging from a mild, self-limited febrile illness to life-threatening disease. Dengue viruses are endemic in most tropical and subtropical regions of the world with more than 2 billion persons at risk for acquiring dengue. For these reasons, the WHO has made development of a dengue vaccine a top priority (Resolution WHA 46.31).

Infection with one dengue serotype induces long-lived homotypic immunity and short-lived heterotypic immunity [3]. Immunity is primarily mediated by neutralizing antibodies against the envelope (E) glycoprotein. Epidemiological studies have demonstrated that most cases of DHF/DSS occur in persons experiencing a second dengue infection with a serotype different from that which caused their first dengue infection [4]. For this reason, DHF/DSS occurs predominately in children or adults living in dengue-endemic regions with multiple dengue serotypes circulating simultaneously. The goal of immunization is to induce a long-lived neutralizing antibody response against all four dengue serotypes. This can be best achieved economically using a live attenuated virus vaccine delivered in one or more doses. Development of a live-attenuated vaccine is a reasonable goal since it has already been achieved for the related yellow fever virus, another mosquito-borne flavivirus present in tropical regions of the world [1].

In humans, dengue virus infects predominately monocytes, dendritic cells, and lymphocytes and does not exhibit tropism for any particular organ, except perhaps the liver [5]. The majority of primary and secondary infections with dengue viruses are asymptomatic. Following an incubation period of approximately one week, self-limiting acute illness designated dengue fever (DF) occurs that is characterized by a febrile period of about 5 days accompanied by systemic symptoms such as headache, malaise, anorexia, arthralgia, and myalgia. Viremia, rash (including petechial hemorrhages), lymphadenopathy, leucopenia, and thrombocytopenia can accompany the fever. Elevations of liver enzymes during DF are common and the virus is known to infect human hepatocytes [6-8]. The virus does not establish persistent infection and is usually eliminated by the end of the second week. DHF/DSS occurs much less commonly than DF. It; it develops at the time of defervescence and is characterized by an increased tendency to bleed into the skin or from mucous membranes and by a marked increase in vascular permeability resulting in hemoconcentration and shock. This state of increased vascular permeability is short-lived (several days) and with proper management is fatal in only about 1% of patients [1]. Because previous

infection with one dengue virus serotype can increase the risk for DHF/DSS following infection with a different serotype, it is clear that a dengue virus vaccine will need to protect against each of the four dengue virus serotypes, namely DEN1, DEN2, DEN3, and DEN4.

The dengue virus genome contains a single open reading frame encoding a polyprotein which is processed by proteases of both viral and cellular origin into 3 structural proteins, namely the capsid (C), membrane, (M) and envelope (E) proteins, and at least 7 non-structural (NS) proteins. Each end of the DEN virus genome consists of an untranslated region (UTR), which is predicted to be highly structured. The E glycoprotein is on the surface of the virion, and immunity is mediated primarily by neutralizing antibodies to this protein. The E protein also defines each of the four DEN virus serotypes (DEN1, DEN2, DEN3, and DEN4). The 3′ untranslated region is highly conserved between the four dengue serotypes. Studies in mice, rhesus monkeys, and mosquitoes were designed to evaluate the level of attenuation conferred independently by chimerization and the Δ30 (30 nucleotide deletion) mutation [9]. The specific objectives of this proposed study are to evaluate the safety, infectivity, and immunogenicity of a 2-dose regimen of the rDEN1Δ30 virus in seronegative adult volunteers to determine this vaccine candidate's suitability for inclusion in a tetravalent dengue vaccine formulation.

2.2 Background – Human Experience with Dengue-1

Numerous studies of wild type DEN1 and live attenuated DEN1 vaccine candidates have been conducted over the past 60 years. Various DEN1 live attenuated vaccine candidate viruses have been administered to nearly 1500 volunteers in Phase 1 and Phase 2 clinical studies [10-17]. The majority of dengue vaccines tested to date have been biologically derived by repeated passage in tissue culture. Although some of these vaccine candidates have appeared promising in early studies, none has achieved the ideal balance between reactogenicity and immunogenicity, particularly when included as part of a tetravalent vaccine formulation [18, 19]. It has been difficult to achieve adequate antibody responses to all four serotypes when the vaccine viruses are administered in the tetravalent formulation. Common adverse events noted in these studies were signs and symptoms of mild dengue fever, notably fever, headache, myalgias, rash, neutropenia, and elevated liver function tests. All volunteers who experienced any adverse event recovered completely and without sequelae. Of note, no live attenuated dengue candidate to date has induced DHF or DSS. The lack of a suitable DEN1 live attenuated vaccine candidate forms the basis for testing the rDEN1Δ30 candidate described in this protocol.

2.3 Vaccine Description

The vaccine candidate rDEN1Δ30 is a live attenuated virus derived from the DEN1 Western Pacific (WP) wild-type strain of dengue using recombinant DNA technology. A full-length cDNA clone of the DEN1 WP genome was constructed and a 30 nucleotide deletion was then created in the 3′ untranslated region. Genome-length, capped, RNA transcripts were synthesized from a SacII linearized plasmid using the AmpliCap SP6 Message Maker Kit (EpiCentre Technologies, Madison, WI)

and purified using the RNeasy Mini Kit (Qiagen, Valencia, CA). Qualified Vero cells were transfected with purified RNA transcripts using 1,2-dioleoyl-3-trimethylammonium-propane liposomal transfection reagent (Roche, Indianapolis, IN), and the cell culture medium was harvested 8 days later. From initial transfection through final amplification, only serum-free medium was used for Vero cell culture and propagation of virus. Reagents used during the transfection process and present initially in fluid harvested from the transfection were diluted more than 10^{12}-fold as a result of the biological cloning (terminal dilution) and amplification of the virus. Following final amplification of the virus, the titer was determined by plaque titration in Vero cells. An aliquot was submitted to Charles River Laboratories (CRL; Malvern, PA) as the seed virus for production of a vaccine pool.

2.3.1 Final Container

The final product is contained in flame-sealed, 1.8 mL cryotubes, containing 0.6 mL (approximately 5.5 log_{10} plaque-forming units [PFU]/mL) Live Recombinant Dengue Virus Type 1 rDEN1Δ30-1545 Vero Grown Virus Vaccine (Lot DEN1#104A).

2.3.2 Composition

The Final Drug Product composition is a concentration of 5.5 log_{10} PFU/mL live recombinant rDEN1Δ30-1545 Dengue Virus Type 1 rDEN1Δ30-1545 Virus Vaccine Lot DEN1#104A in Leibovitz L-15 Medium containing 1X SPG (sucrose, 0.218 M; KH_2PO_4, 0.0038 M; K_2HPO_4, 0.0072 M; mono-sodium glutamate, 0.0054 M).

2.3.3 Investigational Product Label (Enlarged Example)

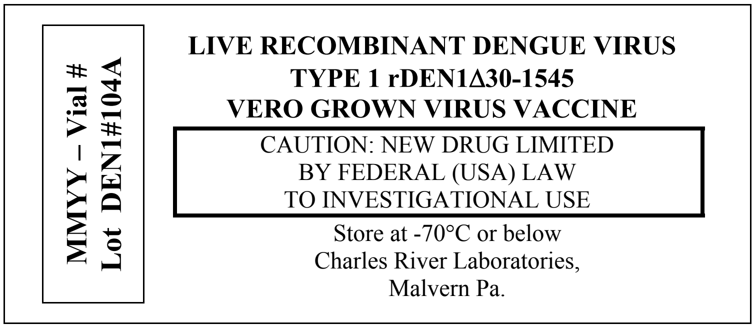

Figure 1: Product Label for Lot DEN1#104A produced by Charles River Laboratories (example)

2.4 Rationale

2.4.1 Animal Experience

The vaccine virus rDEN1Δ30 has been evaluated in juvenile rhesus monkeys and in the novel rodent model consisting of severe combined immunodeficiency (SCID) mice bearing intraperitoneal tumors of the human liver cell line HuH-7. Replication of rDEN1Δ30 virus in the SCID mouse model was compared with that of wild type DEN1 virus and another attenuated DEN1 strain, DEN1mutF. Both the rDEN1Δ30 virus and the DEN1mutF were attenuated in the SCID mice, replicating to a peak titer approximately 100-fold lower than that of wild-type DEN1 [20]. Juvenile rhesus monkeys were inoculated subcutaneously with 10^5 PFU of rDEN1Δ30, DEN1mutF, or wild type DEN1 virus. Monkeys inoculated with wild type DEN1 virus or DEN1mutF virus were viremic for 2 to 3 days and had mean peak virus titers of 2.1 and 1.4 $\log_{10}$ PFU, respectively. In contrast, monkeys inoculated with the candidate vaccine virus rDEN1Δ30 were viremic for less than one day and had a mean peak virus titer of only 0.8 $\log_{10}$ PFU. Despite this high level of attenuation, all rhesus monkeys inoculated with the rDEN1Δ30 virus developed serum neutralizing antibodies against DEN1. Although the mean serum neutralizing titers were lower in those monkeys inoculated with rDEN1Δ30, consistent with its attenuation phenotype, sufficient neutralizing antibody was induced to protect all monkeys against subsequent challenge with wild type DEN1on Study Day 28**.**

2.4.2 Clinical Experience with rDEN1Δ30

A previous lot of the investigational rDEN1Δ30 vaccine was evaluated at a dose of 10^3 PFU in a placebo-controlled Phase 1 clinical study at the Center for Immunization Research at the Johns Hopkins Bloomberg School of Public Health (BSPH) [21]. Twenty-eight healthy male and non-pregnant female adult volunteers, between the ages of 18 and 50, were enrolled in the study; 20 volunteers received vaccine, 8 volunteers received placebo (vaccine diluent). Volunteers were randomly assigned to receive vaccine or placebo.

This vaccine was well tolerated and was found to be both safe and strongly immunogenic. The safety, infectivity, and immunogenicity profile of this vaccine was found to be comparable to rDEN4Δ30 and rDEN2/4Δ30, two other recombinant dengue vaccine candidates (**Tables 2, 3 and 4; Figure 2)** [21]. No vaccinee developed a dengue-like illness at a dose of 10^3 PFU. Local reactogenicity was minimal in all volunteers. The following asymptomatic adverse events were observed in vaccinees receiving rDEN1Δ30 vaccine. One vaccinee developed a Grade 3 creatine phosphokinase (CPK) elevation on Study Day 4 following vaccination. The CPK level returned to normal over a three-day period. One volunteer experienced a low platelet count (116,000/mm^3) on Study Day 16 post-vaccination and returned to normal by Study Day 19. There was no evidence

of bleeding in this volunteer. Eight out of 20 vaccinees experienced a transient neutropenia between Study Days 4 and 16 that resolved in all volunteers. Vaccinated volunteers who had a baseline absolute neutrophil count (ANC) of <3500/mm^3 were more likely to become neutropenic than those individuals whose ANC count at vaccination was >3500/mm^3. Neutropenia was graded as mild in six volunteers (1000–1500/mm_3), moderate in one volunteer (750–999), and severe in one volunteer (500 –749). None of the neutropenic volunteers developed clinical complications. No volunteer experienced an elevation in liver function tests. Finally, 40% of vaccinees developed a faint maculopapular rash over the trunk and proximal upper extremities. The rash was asymptomatic and lasted approximately one week.

Nine of twenty volunteers who received vaccine had detectable vaccine virus in the blood **(Table 2)**. The mean peak virus titer was 1.0 $\log_{10}$ PFU/mL, which was slightly higher than that observed in volunteers who received 10^3 PFU of the live attenuated rDEN4Δ30 vaccine. Ninety-five percent of vaccinated volunteers receiving rDEN1Δ30 seroconverted to DEN1 by Study Day 42, as defined by a ≥4-fold rise in serum neutralizing antibody **(Table 3)**. All vaccinees who seroconverted to rDEN1Δ30 maintained excellent antibody titers out to Study Day 180.

Table 2: Magnitude, Onset, and Duration of Viremia in Volunteers Inoculated with 10^3 PFU of rDEN1Δ30, rDEN2/4Δ30, or rDEN4Δ30

Vaccine Candidate	Dose (log_{10} PFU)	No. Volunteers	No. with Viremia	Mean Peak Titer ± SE[a] (log_{10} PFU/mL) [Stat Group][c]	Peak Viremia Range[b] (log_{10} PFU/mL)	Mean Day of Viremia Onset ± SE [Stat Group]	Mean no. Days of Viremia ± SE [Stat Group]
DEN1Δ30	3.0	20	9	1.0 ± 0.2[A]	0.5 – 1.9	9.8 ± 0.7 [A]	2.8 ± 0.8 [A]
DEN2/4Δ30	3.0	20	11	0.6 ± 0.1 [A]	0.5 – 1.2	9.1 ± 1.0 [A]	3.2 ± 0.7 [A]
DEN4Δ30[d]	3.0	20	7	0.5 ± 0.0 [B]	0.5 ± 0	9.1 ± 0.9 [A]	1.6 ± 0.6 [A]

[a] Mean peak titer is calculated only for those volunteers who were viremic.
[b] Peak viremia range is reported only for those volunteers with detectable viremia.
[c] Groups not assigned the same letter are significantly different ($\alpha = 0.05$).
[d] Not tested.

Table 3: Serum Antibody Response Induced by 10^3 PFU of rDEN1Δ30, rDEN2/4Δ30, or rDEN4Δ30

Vaccine Candidate	Dose (log_{10} PFU)	No. of Volunteers	No. Infected[a]	Geometric Mean Serum Neutralizing Antibody Titer (Range)			% Seroconversion[c]
				Day 28[b]	Day 42	Day 180	
rDEN1Δ30	3.0	20	19	444 (<10 – 6309)	331 (<10 – 2753)	257 (11 – 1043)	95
DEN2/4Δ30	3.0	20	20	147 (11– 1043)	237 (42 – 3997)	84 (19 – 317)	100
rDEN4Δ30[d]	3.0	20	20	139 (<10 – 2365)	129 (12 – 1222)	NT[d]	95

[a] Defined as either recovery of vaccine virus from the blood or by seroconversion.
[b] $PRNT_{60}$ was <10 for all volunteers on Study Day 0.
[c] Defined as a ≥4-fold rise in serum neutralizing titer for homologous DEN serotype, which corresponds to a $PRNT_{60}$ ≥40.
[d] Not tested.

Table 4: Clinical Summary of rDEN1Δ30, rDEN2/4Δ30(ME), and rDEN4Δ30 Candidate Vaccines

Vaccine	Dose (log_{10} PFU)	No. of Subjects	No. with Viremia	Mean Peak Titer (log_{10})	Clinical Signs (No. of Volunteers with Indicated Clinical Sign)			
					Fever	Rash	Neutropenia	↑ALT
rDEN1Δ30	3	20	9	1.0	1	8	8	0
rDEN2/4Δ30(ME)	3	20	11	0.6	0	9	7	3
rDEN4Δ30	3	20	7	0.5	0	11	5	1

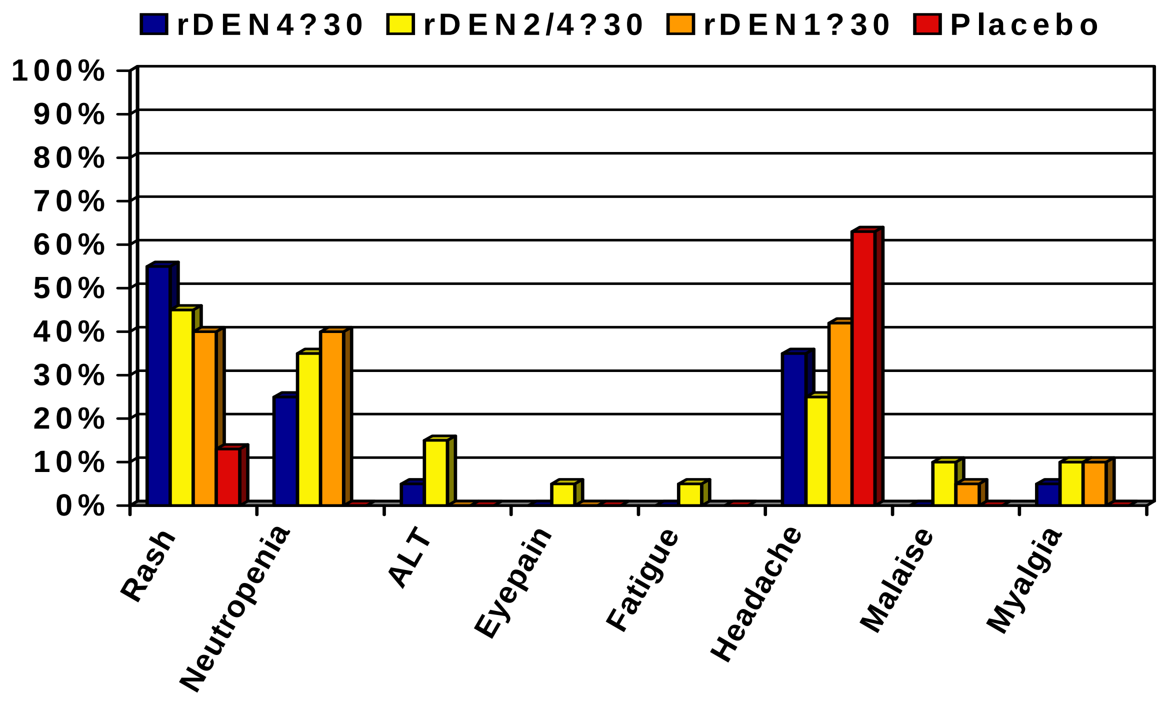

Figure 2: Solicited Adverse Events Recombinant Dengue Vaccines

2.5 Mosquito Transmissibility

Aedes aegypti and *A. albopictus* were fed a blood meal containing serial dilutions of virus suspension [20]. They were maintained for 21 days and then frozen. Head and midgut preparations were processed for immunofluorescence assays to determine the presence of viral antigen. Both the wt rDEN1 and rDEN1Δ30 demonstrated a low infectivity (mosquito infectious dose$_{50}$ [MID$_{50}$] of >10^3 PFU/mL). This is consistent with previously reported studies which demonstrated a very low infectivity of DEN1 for *A. aegypti* fed an infectious blood meal. It is unlikely that the vaccine virus would be transmitted from an infected vaccinee to a mosquito since rDEN1Δ30 is poorly infectious for mosquitoes. In addition, if a similar low level of viremia is seen in humans as in monkeys, rDEN1Δ30 should be poorly transmitted to mosquitoes, as previously observed for the attenuated rDEN4Δ30 vaccine candidate, which was not transmitted from 10 infected vaccinees to over 300 feeding mosquitoes [22].

2.6 Clinical Development Plan

The rDEN1Δ30 vaccine study will be carried out over approximately 46 weeks in the Baltimore-Washington area. The study will be a double-blinded, placebo-controlled Phase 1 study in healthy adult male and non-pregnant female volunteers aged 18 – 50 years old. The purpose of the study is to evaluate the safety and immunogenicity of a 2-dose regimen of the live attenuated rDEN1Δ30 candidate vaccine in flavivirus-naïve individuals and to determine the optimal boost schedule. The dose of 10^3 PFU was chosen based on the clinical experience with the rDEN1Δ30 vaccine which was found to infect 95% of volunteer when given at a dose 10^3 PFU in the previous Phase 1 study. In cohort 1, 25 vaccinees will receive 10^3 PFU of the rDEN1Δ30 vaccine and 5 volunteers will receive placebo as a single subcutaneous injection on Study Days 0 and 120. In cohort 2, 25 vaccinees will receive 10^3 PFU of the rDEN1Δ30 vaccine and 5 volunteers will receive placebo as a single subcutaneous injection on Study Days 0 and 180. Volunteers will be randomly assigned to receive vaccine or placebo.

2.7 Participation of Children

Although this vaccine has been evaluated in 20 healthy adults and tolerated well, it is felt that insufficient data are available to judge the potential risk in children. Once safety is established in adults in the United States and then in endemic regions, we hope to extend vaccination to children in dengue-endemic regions.

2.8 Statement of Compliance

This study will be conducted in compliance with the protocol, Good Clinical Practice guidelines, Food and Drug Administration (FDA) regulations, and the Western Institutional Review Board (WIRB).

3.0 OBJECTIVES

3.1 Primary Objectives

To determine safety and immunogenicity of a 2-dose regimen of the rDEN1Δ30 vaccine with the second dose given at Study Day 120 or 180 by

- Determining the frequency of vaccine related AEs for each dose graded by severity.
- Comparing the immunogenicity of the two 2-dose regimens of the rDEN1Δ30 candidate vaccine as assessed by neutralizing antibody titers to DEN1 at 4 and 6 weeks after first and second vaccination.

3.2 Secondary Objectives

- To assess the frequency, quantity, and duration of viremia after each dose of vaccine.
- To determine the number of vaccinees infected with rDEN1Δ30. Infection is defined as recovery of vaccine virus from the blood or serum of a volunteer and/or by seroconversion to DEN1 (a ≥4-fold rise in DEN1 neutralizing antibody titers).
- To compare the infectivity rates, safety, and immunogenicity between dose 1 and dose 2 within a cohort and between cohorts.
- To evaluate the immunopathological mechanism of vaccine-associated rash in those volunteers who are willing to undergo skin biopsy.
- To evaluate the phenotype and activation of peripheral blood mononuclear cells (PBMCs) at primary infection and challenge with DEN1.

4.0 STUDY DESIGN

4.1 Overall Design

The study is a placebo-controlled, double-blind study in normal healthy adult volunteers recruited from the metropolitan Baltimore/Washington area. The purpose of this study is to evaluate the safety, reactogenicity, and immunogenicity of two doses of the live attenuated dengue 1 vaccine virus rDEN1Δ30. A placebo arm is included in the study as a control to better assess vaccine-associated versus non vaccine-associated adverse events. After providing written informed consent, volunteers will undergo eligibility screening, including medical history, physical examination, hematology testing, liver and renal function testing, human immunodeficiency virus (HIV), hepatitis B and C screening, urinalysis, and serology screening for previous flavivirus infection. Urine pregnancy testing will be performed on female volunteers. All screening tests must be performed within 60 days of vaccination. All clinically significant abnormalities will be reviewed with volunteers and referral for follow-up care will be provided. Volunteers will be determined to be eligible based on the inclusion and exclusion criteria found in **Section 5.0** of this protocol. For participants who are eligible, the Day 0 visit will be scheduled to receive the first vaccination. Urine pregnancy testing will be repeated on female volunteers on the day of each vaccination and all volunteers will undergo

repeat hepatitis B, hepatitis C, and HIV studies prior to second vaccination. Cohort 1 will return for a second vaccination at Study Day 120, cohort 2 will return for a second vaccination at Study Day 180. Because this is a live vaccine, volunteers will be re-screened for HIV, hepatitis C, and hepatitis B prior to administration of the second dose of vaccine. After each vaccination, volunteers will be evaluated in the clinic for at least 30 minutes and then approximately every other day for the first 16 days of the study. They will have blood drawn for clinical laboratory studies, virologic assays, and immunologic assays. They will also have a clinical evaluation performed at each visit (see Section 7.4 for detailed description of study procedures). They will return to the clinic on Study Days 21, 28, and 42 post-vaccination for clinical evaluation and blood draw. All volunteers will complete a diary card on which they will record their temperature three times a day for the first 16 days post-vaccination.

4.2 Dosing Strategy

Thirty volunteers will be enrolled in cohort 1 and will receive either 10^3 PFU of rDEN1Δ30 vaccine (25 volunteers) or placebo (5 volunteers) on Study Days 0 and 120. Thirty volunteers will be enrolled in the cohort 2 and will receive either 10^3 PFU of rDEN1Δ30 vaccine (25 volunteers) or placebo (5 volunteers) on Study Days 0 and 180. Volunteers in both cohorts may be enrolled simultaneously at the discretion of the investigator.

4.3 Sample Size and Estimated Duration of the Study

Sixty volunteers (50 vaccinees and 10 placebo recipients) will be enrolled in the study.

Twenty-five volunteers in each dose cohort will receive study vaccine and 5 volunteers will receive placebo. Twenty-five volunteers were chosen to receive vaccine in each cohort for the following reasons:

- This study is designed to build upon the previous Phase 1 study of rDEN1Δ30. Safety, virologic, and immunologic data after the first dose of vaccine will be pooled with historical data.
- The ability of the second dose of vaccine to boost the antibody response will be easily detectable in the small number of volunteers chosen. If the second dose of vaccine is unable to boost the antibody response, this too will be apparent in a small number of volunteers.
- We have included placebo-recipients in each treatment arm so that we can better assess if common adverse events such as headache, myalgia, and arthralgia are vaccine-related. Placebo recipients from both cohorts can be combined in the final analysis for a better assessment of which adverse events are more common in vaccine recipients and which are not. For this reason, we have reduced the number of placebo recipients in each cohort to 5, reducing the total numbers of volunteers required to meet the study objectives.

Volunteers in cohort 1 will be followed for approximately 23 weeks (approximately 162 days) from the time of their first vaccination. Volunteers in cohort 2 will be

followed for approximately 32 weeks (approximately 222 days) from the time of their first vaccination.

4.4 Treatment Assignment

Volunteers will know if they are in cohort 1 or cohort 2. Volunteers within each cohort will be randomly assigned to receive rDEN1Δ30 vaccine or placebo. Treatment assignment will be performed using a random number generator by personnel who have no involvement in the clinical assessment of volunteers. A master log of treatment assignment will be maintained in a record separate from other study records. This log will be kept in the Center for Immunization Research laboratory in the School of Public Health. It will be kept in a locked room with limited access. A sealed envelope containing a copy of the treatment assignment will also be kept by the Data and Safety Monitoring Board (DSMB) executive secretary, and a representative of Science Applications International Corporation, Regulatory Compliance Human Subjects Protection Program. The volunteers will be informed of their treatment assignment after the study has been unblinded (Day 42 post-second vaccination for all volunteers in the respective cohort).

4.5 Blinding

This study will be conducted as a double-blind study to avoid biased assessment of adverse events. Vaccine or placebo will be prepared and drawn up into syringes by laboratory personnel who are not involved with the clinical assessment of volunteers. Syringes are labeled with volunteer number only. Because vaccine diluent is used as placebo, there will be no difference in the appearance of the syringes. The volunteer, investigator, and clinical staff will not know to which treatment group the volunteer has been assigned. In addition, other personnel assigned to monitor the study will not know the treatment assignment of the volunteer. Clinical staff will remain blinded to treatment assignment until all volunteers within a cohort have reached Study Day 42 post-second vaccination. If the need arises to unblind a specific volunteer's assignment for emergency medical management prior to all volunteers completing Study Day 42 post second vaccination, the Principal Investigator will contact Center for Immunization Research (CIR) laboratory personnel and obtain the treatment assignment of the volunteer in question. Only that specific volunteer's assignment will be unblinded. The Sponsor and the DSMB executive secretary will be notified of the event within two business days.

4.6 Duration of Participation

Duration of individual volunteer participation in the study is approximately 23 weeks (162 days) for those volunteers in cohort 1 and approximately 32 weeks (222 days) for those volunteers in cohort 2. The entire duration of the study is approximately 46 weeks.

5.0 SELECTION AND ENROLLMENT OF VOLUNTEERS

5.1 Inclusion Criteria

1. Adult males and non-pregnant, non-breastfeeding females between 18 and 50 years of age, inclusive. Children will not be recruited or enrolled in this study for safety considerations.
2. Good general health as determined by means of the screening procedures.
3. Available for the duration of the study (23 weeks for cohort 1 and 32 weeks for cohort 2).
4. Willingness to participate in the study as evidenced by signing the informed consent document.

5.2 Exclusion Criteria

1. Pregnancy as determined by a positive urine human choriogonadotropin (β-HCG) test (if female).
2. Currently lactating and breast-feeding (if female).
3. Participant is unwilling to use reliable contraception methods for the duration of the study (Reliable methods of birth control include: pharmacologic hormonal contraceptives including oral, parenteral, and transcutaneous delivery; condoms with spermicide, diaphragm with spermicide, surgical sterilization, intrauterine device, abstinence, and post-menopause.)
4. Evidence of clinically significant neurologic, cardiac, pulmonary, hepatic, rheumatologic, autoimmune, or renal disease by history, physical examination, and/or laboratory studies including urinalysis.
5. Behavioral, cognitive, or psychiatric disease that in the opinion of the investigator affects the ability of the volunteer to understand and cooperate with the study protocol.
6. Neutropenia as defined by an absolute neutrophil count $<1500/mm^3$.
7. Alanine transaminase (ALT) level above the laboratory-defined upper limit of normal.
8. Serum creatinine level above the laboratory-defined upper limit of normal.
9. Other condition that in the opinion of the investigator would jeopardize the safety or rights of a volunteer participating in the study or would render the volunteer unable to comply with the protocol.
10. Volunteer has had medical, occupational, or family problems as a result of alcohol or illicit drug use during the past 12 months.
11. History of a severe allergic reaction or anaphylaxis.
12. Severe asthma (requiring an emergency room visit or hospitalization within the last 6 months).
13. Positive HIV-1 serology by screening and confirmatory assays.
14. Positive for hepatitis C virus (HCV) by screening and confirmatory assays.
15. Positive hepatitis B surface antigen (HBsAg) by enzyme-linked immunosorbent assay (ELISA).

16. Known immunodeficiency syndrome.
17. Use of immunosuppressive doses of corticosteroids (excluding topical or nasal) or immunosuppressive drugs within 30 days prior to vaccination. Immunosuppressive dose of corticosteroids is defined as ≥ 10mg prednisone equivalent per day for ≥ 14 days.
18. Receipt of a live vaccine within the past 4 weeks or a killed vaccine within the past 2 weeks prior to entry into the study.
19. History of a surgical splenectomy.
20. Receipt of blood products within the past 6 months.
21. History or serologic evidence of previous dengue virus infection or other flavivirus infection (yellow fever virus, St. Louis encephalitis, West Nile virus).
22. Previous receipt of yellow fever or dengue vaccine (licensed or experimental).
23. Persons who have definite plans to travel to a dengue endemic area during the study will be excluded from the study.
24. Persons who have received an investigational agent in the past 30 days.

5.3 Access of Medical Records

The medical history of a volunteer will be obtained directly from the volunteer. No medical records will be requested unless there is a need to clarify the volunteer's medical history. No medical record will be requested without the informed written consent of the volunteer.

5.4 Treatments that Could Potentially Interfere with Vaccine-Induced Immunity

The following criteria will be checked at on day of vaccination and on study days 28 and 42 following each vaccination. If any become applicable during the study, the participant will not be included in the immunogenicity evaluations after the time of exclusion. The participant will, however, be encouraged to remain in the study for safety evaluation for the duration of the study.

1. Use of any investigational drug or investigational vaccine other than the study article during the study period.
2. Chronic administration (≥14 days) of steroids (defined as prednisone equivalent of 10 mg per day), immunosuppressants or other immune-modifying drugs during the 28-day period post-vaccination 1 or 2, or during the 28-day period prior to vaccination 2. (Topical and nasal steroids are allowed.)
3. Receipt of a licensed vaccine from Study Day 0 to Study Day 28 and within 28 days after the second dose of the investigational vaccine.
4. Receipt of immunoglobulins and/or any blood products from Study Day 0 until 28 days after the second dose of the investigational vaccine.

In addition, if a volunteer should become pregnant during the study, the participant will not be included in the immunogenicity evaluations from that point forward. She

will, however, be encouraged to remain in the study for periodic safety evaluations and will be followed until completion of her pregnancy. The volunteer will be asked to sign a release of medical information form so that records can be obtained from her obstetrician regarding the outcome of the pregnancy.

5.5 Volunteer Withdrawal/Termination Criteria

A volunteer will not be considered to have completed the study if any of the following reasons listed below apply. However, any volunteer who has received vaccine or placebo will be encouraged to remain in the study for periodic safety evaluations for the duration of the study. In the event that a volunteer becomes incarcerated during the course of the study, every attempt will be made to contact the volunteer for determination of his/her safety. Once a safety assessment had been made on an incarcerated volunteer, he/she may be terminated from the study if his/her period of incarceration will make him/her unable to make scheduled visits.

1. *Research terminated by Sponsor or investigator* – applies to the situation where the entire study is terminated by the Sponsor or Principal Investigator for any reason.
2. *Withdrawal of consent* – applies to a volunteer who withdraws their consent to participate in the study for any reason.
3. *Noncompliant with protocol* – applies to a volunteer who does not comply with protocol-specific visits or evaluations, on a consistent basis, such that adequate follow-up is not possible and the volunteer's safety would be compromised by continuing in the study. Additionally, this applies to a volunteer who is lost to follow-up and is not reachable by telephone or other means of communication, and therefore not able to be located. In the event that a volunteer becomes incarcerated during the course of the study, and it is determined that he or she is unable to participate in the study, he or she will be terminated from the study.
4. *Volunteer withdrawal* may occur if the investigator believes that it is in the best interest of the volunteer to be withdrawn from the study.
5. *Other* – is a category used when previous categories do not apply and requires an explanation.

5.6 Exclusion from Receipt of Second Vaccination

A volunteer will not receive a second vaccination if any of the following conditions is met:

1. Positive HIV-1 serology by screening and confirmatory assays tested within one month of second vaccination.
2. Positive for hepatitis C virus (HCV) by screening and confirmatory assays tested within one month of second vaccination.
3. Positive hepatitis B surface antigen (HBsAg) by enzyme-linked immunosorbent assay (ELISA) tested within one month of second vaccination.
4. Positive urine pregnancy test on day of second vaccination (females only).
5. Receipt of a live vaccine within the 4 weeks prior to or a killed vaccine within the 2 weeks prior to second vaccination.

6. Use of immunosuppressive doses of corticosteroids (excluding topical or nasal) or immunosuppressive drugs within 30 days prior to second vaccination. Immunosuppressive dose of corticosteroids is defined as ≥ 10mg prednisone equivalent per day for ≥ 14 days.
7. Persons who have received an investigational agent in the 30 days prior to second vaccination.
8. History of severe allergic reaction or anaphylaxis associated with first vaccination.

5.7 Replacement of Volunteers

Volunteers who have been excluded from evaluation of immunogenicity, terminated from the study or who have withdrawn their consent can only be replaced if they have left the study prior to providing the Day 42 blood sample following the first vaccination. If a volunteer is terminated from the study after Day 42 but prior to the second vaccination, the volunteer will not be replaced. Analysis of seroconversion rates at the Study Day 42 post second vaccination time-point will include only those volunteers who are still enrolled on Day 42 post-second vaccination. Replacement volunteers will be assigned a study number different from that of the volunteer they are replacing.

6.0 VACCINE PREPARATION

6.1 Supplies

Vaccine virus for this protocol will be stored at the NIAID-designated cGMP storage facility, repository, until requested by the CIR. Vials of frozen vaccine for administration to volunteers will be formally requested to be transferred to the CIR laboratories at the Johns Hopkins Bloomberg School of Public Health by the Principal Investigator after IRB approval and FDA review of the study has been completed. Vaccine will then be stored in a locked –70°C freezer at the CIR laboratories until time of use. After a vaccine shipment is received by the CIR laboratories, a sample (i.e., several vials) of vaccine will be tested for titer and stability per standard laboratory protocols. The vaccine is supplied as a concentrate that must be diluted to the proper dose prior to administration. Prior to vaccination, the Principal Investigator will supply the laboratory with a test article request form which will include the protocol number, the vaccine virus name, the vaccine titer (concentration), the IND number, the number of volunteers to be vaccinated and the dilution instructions for the vaccine. Laboratory personnel will prepare the correct dose of vaccine (or placebo) for each volunteer in a biosafety hood using sterile technique. Vaccine will be diluted as described in the respective standard operating procedure with Leibovitz–15 (L-15) medium from a specific lot tested for sterility, general safety, and identity as described in the RCHSPB-sponsored BB-MF 12959. The diluted vaccine (or placebo) will be drawn up to a volume of 0.5 mL in a 1 mL syringe and labeled with the volunteer number. The labeled syringes will be transported on wet ice to the clinic for administration. Vaccine must be used within four hours of being removed from the freezer.

The vaccine diluent, 1X Leibovitz L-15 medium is also used as the placebo. The 1X Leibovitz L-15 medium is prepared from reagents that have undergone additional quality testing prior to use. A concentrated form, 2X, of Leibovitz L-15 medium is mixed 1:1 with sterile water for injection. A Type II Master File for 2X Leibovitz L-15 medium, BB-MF 12959, has been created and submitted to the FDA that contains the testing results for the currently used lot of 2X Leibovitz L-15 Medium.

6.2 Vaccine Storage

The rDEN1Δ30 vaccine should remain frozen at –70°C until just prior to use. Vaccine should never be refrozen for reuse in volunteers. Diluent components may be stored at 2°C - 8°C (2X Leibovitz L-15 medium) or room temperature (sterile water for injection) per manufacturer's recommendation. Vaccine and diluent components must be taken from new, sealed containers for each use. No component should be reused for vaccine or placebo preparation.

6.3 Vaccine Accountability

CIR vaccine preparation personnel will maintain an accurate inventory and accountability record of vaccine supplies for this study. Partially used vials of vaccine or placebo components may not be administered to other volunteers.

6.4 Disposition of Used/Unused Supplies

After CIR laboratory personnel have diluted the vaccine and drawn up the syringes for administration, they will remove the label from the vaccine vial and place it in the accountability log. In this manner, monitoring personnel will be able to verify the accountability of all vaccine vials used for the study. An aliquot of undiluted (if available) and diluted vaccine will be titered by laboratory personnel after vaccine has been prepared and delivered to the clinical staff. This is done to confirm the titer of vaccine administered to the volunteers. An aliquot of diluted vaccine will also be frozen and stored at –70°C in the CIR laboratory in case it needs to be re-titered at a later time. If there is any vaccine left after the syringes have been drawn up and an aliquot removed for titering, it will be destroyed by the laboratory personnel. Any unused diluent/placebo will also be destroyed.

7.0 STUDY PROCEDURES

The following sections provide a detailed listing of the procedures and studies to be performed in this protocol at designated time points. The total volume of blood (approximately 700 mL) to be drawn over the duration of the study (4.5 or 6.5 months) is about the same as donating a unit and a half of blood. This is well within guidelines of blood donation and should not compromise the health of study participants.

7.1 Recruitment and Screening

Volunteers will be recruited using a variety of sources, including volunteers previously enrolled in vaccine studies at the CIR; the use of a center-wide IRB approved screening protocol; and by the use of study-specific IRB-approved print and/or other media advertising. After an initial screen (included in supplements to the protocol) by clinical staff consisting of background information of the study and a review of basic inclusion and exclusion criteria, a screening visit will be scheduled. During the study-specific screening, which may require more than one visit, the volunteer will read the consent form, be encouraged to ask questions, and then take a multiple-choice questionnaire to evaluate consent comprehension (included in supplements to the protocol). Study staff will use incorrect answers from the questionnaire to identify those areas of the informed consent form that need further review with the volunteer. The volunteer must answer all questions correctly prior to being eligible for enrollment. This will help ensure the volunteer has sufficient understanding before the consent form is signed. The volunteer may either sign the consent form during the screening visit or sign it after further consideration.

7.2 Screening Procedures (Up to 60 Days Prior to First Vaccination)

1. Explain the study and Informed Consent to the volunteer.
2. Ensure that the volunteer has successfully completed the Informed Consent comprehension assessment, has signed the Informed Consent, and receives a signed copy of the Informed Consent.
3. Explain the HIV Informed Consent and ensure that the volunteer has signed the HIV Informed Consent.
4. Elicit a complete medical history, including menstrual and contraceptive history and/or history of surgical sterility for female volunteers.

5. Perform a complete physical examination.
6. Obtain approximately 30 mL of blood for hematology, biochemistry, serologic tests for viral hepatitis B and C, HIV, and flavivirus infection.
7. Obtain urine for urine dipstick testing (glucose, bilirubin, ketones, specific gravity, blood, pH, protein, urobilinogen, nitrite, leukocyte esterase), as well as β-HCG testing in females.
8. Counsel females to avoid becoming pregnant during the study.

7.3 Immunization Procedure

Volunteers will receive one immunization on Study Day 0 and a second immunization either on Study Day 120 or Study Day 180. Vaccine will be kept frozen at –70°C until just before use where upon it will be thawed and diluted (see Section 6.1 for vaccine preparation). Vaccine or placebo will be kept on wet ice from the time it is diluted until it is delivered to clinical staff for administration. Vaccine or placebo (0.5 mL) will be delivered by subcutaneous injection in the deltoid region of the upper arm with a needle of appropriate gauge and length after preparation of the site with alcohol.

7.4 Clinical Monitoring and Evaluation

Study procedures performed at each visit are listed below. Photographs may be taken of the injection site. In addition, photographs may be taken of other areas of the skin to follow any rash that may develop. The total amount of blood to be drawn throughout the 6-month duration of the study, including screening, is approximately 700 mL.

FIRST VACCINATION

The following visits will occur after vaccination #1 for both cohorts 1 and 2

Study Day 0 (Day of Vaccination)

1. Verify that Informed Consent was obtained.
2. Verify that all applicable eligibility criteria have been met.
3. Perform interim history and focused physical exam, concentrating on any acute complaints.
4. Obtain approximately 35 mL of blood for complete blood count (CBC) with differential, prothrombin time/partial thromboplastin time (PT/PTT), ALT, virology, and immunology (antibody and cellular immune studies).
5. For females, obtain a urine sample for β-HCG testing. Ensure the test is negative before proceeding; a positive test will exclude the volunteer from the study.
6. Record vital signs (blood pressure, temperature, heart rate, and respiratory rate).
7. Administer the vaccine.
8. Observe for at least 30 minutes after vaccination and evaluate for immediate adverse reactions.

9. Education by study staff during the 30 minute post-immunization waiting period describing proper use of digital thermometers, the proper method of filling out the diary card, signs and symptoms of potential adverse events, and how and when to contact study staff.

Study Day 2

1. Perform interim history and focused physical exam emphasizing examination of any acute complaints.
2. Record vital signs.
3. Obtain approximately 15 mL of blood for CBC with differential and virology.

Study Day 4 (± 1 day)

1. Perform interim history and focused physical exam emphasizing examination of any acute complaints.
2. Record vital signs.
3. Obtain approximately 25 mL of blood for CBC with differential, PT/PTT, ALT, and virology.

Study Day 6 (± 1 day)

1. Perform interim history and focused physical exam, emphasizing examination of any acute complaints.
2. Record vital signs.
3. Obtain approximately 20 mL of blood for CBC with differential, ALT, and virology.

Study Day 8 (± 1 day)

1. Perform interim history and focused physical exam, emphasizing examination of any acute complaints.
2. Record vital signs.
3. Obtain approximately 35 mL of blood for CBC with differential, PT/PTT, ALT, virology, and immunology (humoral and cellular immune studies).

Study Day 10 (± 1 day)

1. Perform interim history and focused physical exam, emphasizing examination of any acute complaints.
2. Record vital signs.
3. Obtain approximately 20 mL of blood for CBC with differential, ALT and virology.

Study Day 12 (± 1 day)

1. Perform interim history and focused physical exam, emphasizing examination of any acute complaints.
2. Record vital signs.
3. Obtain approximately 25 mL of blood for CBC with differential, PT/PTT, ALT, and virology.

Study Day 14 (± 1 day)

1. Perform interim history and focused physical exam, emphasizing examination of any acute complaints.
2. Record vital signs.
3. Obtain approximately 40 mL of blood for CBC with differential, ALT, virology, and immunology (humoral and cellular immune studies).

Study Day 16 (± 1 day)

1. Perform interim history and focused physical exam, emphasizing examination of any acute complaints.
2. Record vital signs.
3. Obtain approximately 20 mL of blood for CBC with differential, PT/PTT, ALT, and virology.

Study Day 21 (± 1 day)

1. Perform interim history and focused physical exam, emphasizing examination of any acute complaints.
2. Record vital signs.
3. Obtain approximately 15 mL of blood for ALT, and immunology (antibody and cellular immune studies). Hematology will be done if prior WBC, ANC, platelet count, or hemoglobin results are outside of the normal laboratory ranges. Virology will be done on volunteers who had vaccine virus recovered from the blood on Study Day 16 and will be repeated at each visit until viremia has cleared.
4. Collect diary card.

Study Day 28 (± 2 days)

1. Perform interim history for interim complaints and receipt of treatment that could interfere with immune assessment.
2. Record vital signs.
3. Obtain approximately 25 mL of blood for immunology (antibody and cellular immune studies) and PT/PTT. ALT will be done if indicated (if values from Study Day 21 were abnormal and clinically significant).

Study Day 42 (± 2 days)

1. Perform interim history for interim complaints and receipt of treatment that could interfere with immune assessment.
2. Record vital signs.
3. Obtain approximately 20 mL of blood for immunology (antibody and other immunological assays).

SECOND VACCINATION

Volunteers will be retested for HIV antibody, HCV antibody, and Hepatitis B surface antigen in the 30-day period prior to second vaccination. Female volunteers will also have a urine pregnancy test performed in the 30-day period prior to vaccination. Volunteers with positive HIV-1, HCV, or hepatitis B serology by screening and confirmatory assays will be excluded from second vaccination. Female volunteers with a positive urine β-hCG test will be excluded from second vaccination.

Cohort 1 Only

Study Day 90 (+ 44 days but within 30 days of second vaccination)
1. Explain the HIV Informed Consent and ensure that the volunteer has signed the HIV Informed Consent.
2. Obtain approximately 10 mL of blood for serologic tests for viral hepatitis B and C, and HIV.
3. Repeat urine pregnancy test (female volunteers only).
4. Review pregnancy prevention with female volunteers.

Study Day 120 (+ 14 days)
1. Perform interim history and focused physical exam, concentrating on any acute complaints.
2. Ensure that identified exclusionary criteria for second vaccination have not been met (Section 5.6)
3. Obtain approximately 35 mL of blood for CBC with differential, PT/PTT, ALT, virology, and immunology (humoral and cellular immune studies).
4. For females, obtain a urine sample for β-HCG testing. Ensure that the test is negative before proceeding; a positive test will exclude the volunteer from further vaccinations.
5. Record vital signs (blood pressure, temperature, heart rate, and respiratory rate).
6. Administer the vaccine.
7. Observe for at least 30 minutes after vaccination and evaluate for immediate adverse reactions.
8. Education by study staff during the 30 minute post-immunization waiting period describing proper use of digital thermometers, the proper method of filling out diary card, signs and symptoms of potential adverse events, and how and when to contact study staff.

Study Day 122 (Day 2 post second vaccination)
1. Perform interim history and focused physical exam, emphasizing examination of any acute complaints.
2. Record vital signs.
3. Obtain approximately 15 mL of blood for CBC with differential, and virology.

Study Day 124 (Day 4 post second vaccination ± 1 day)

1. Perform interim history and focused physical exam, emphasizing examination of any acute complaints.
2. Record vital signs.
3. Obtain approximately 25 mL of blood for CBC with differential, PT/PTT, ALT, and virology.

Study Day 126 (Day 6 post second vaccination ± 1 day)

1. Perform interim history and focused physical exam, emphasizing examination of any acute complaints.
2. Record vital signs.
3. Obtain approximately 20 mL of blood for CBC with differential, ALT, and virology.

Study Day 128 (Day 8 post second vaccination ± 1 day)

1. Perform interim history and focused physical exam, emphasizing examination of any acute complaints.
2. Record vital signs.
3. Obtain approximately 35 mL of blood for CBC with differential, PT/PTT, ALT, virology, and immunology (humoral and cellular immune studies).

Study Day 130 (Day 10 post second vaccination ± 1 day)

1. Perform interim history and focused physical exam, emphasizing examination of any acute complaints.
2. Record vital signs.
3. Obtain approximately 20 mL of blood for CBC with differential, ALT, and virology.

Study Day 132 (Day 12 post second vaccination ± 1 day)

1. Perform interim history and focused physical exam, emphasizing examination of any acute complaints.
2. Record vital signs.
3. Obtain approximately 25 mL of blood for CBC with differential, PT/PTT, ALT, and virology.

Study Day 134 (Day 14 post second vaccination ± 1 day)

1. Perform interim history and focused physical exam, emphasizing examination of any acute complaints.
2. Record vital signs.
3. Obtain approximately 40 mL of blood for CBC with differential, ALT, virology, and immunology (humoral and cellular immune studies).

Study Day 136 (Day 16 post second vaccination ± 1 day)

1. Perform interim history and focused physical exam, emphasizing examination of any acute complaints.
2. Record vital signs.
3. Obtain approximately 25 mL of blood for CBC with differential, PT/PTT, ALT, and virology.

Study Day 141 (Day 21 post second vaccination ± 1 day)

1. Perform interim history and focused physical exam, emphasizing examination of any acute complaints.
2. Record vital signs.
3. Obtain approximately 15 mL of blood for ALT and immunology (antibody and cellular immune studies). Hematology will be done if day 136 WBC, ANC, platelet count, or hemoglobin results are outside of the normal laboratory ranges. Virology will be done on volunteers who had vaccine virus recovered from the blood on Study Day 136 and will be repeated at each visit until viremia has cleared.
4. Collect diary card.

Study Day 148 (Day 28 post second vaccination ± 2 days)

1. Perform interim history of complaints, including history of receipt of treatment that could interfere with immune assessment.
2. Record vital signs.
3. Obtain approximately 25 mL of blood for immunology (antibody and cellular immune studies) and PT/PTT. ALT will be done if indicated (if values from Study Day 141 were abnormal and clinically significant).

Study Day 162 (Day 42 post second vaccination ± 2 days)

1. Perform interim history of complaints, including receipt of treatment that could interfere with immune assessment.
2. Record vital signs.
3. Obtain approximately 20 mL of blood for immunology (antibody and other immunological assays).

Cohort 2 Only

Study Day 150 (+ 44 days but within 30 days of second vaccination)

1. Explain the HIV Informed Consent and ensure that the volunteer has signed the HIV Informed Consent.
2. Obtain approximately 10 mL of blood serologic tests for viral hepatitis B and C, and HIV.
3. Repeat urine pregnancy test (female volunteers only).
4. Review pregnancy prevention with female volunteers.

Study Day 180 (+ 14 days)

1. Perform interim history and focused physical exam, concentrating on any acute complaints.
2. Obtain approximately 35 mL of blood for CBC with differential, PT/PTT, ALT, virology, and immunology (antibody and cellular immune studies).
3. For females, obtain a urine sample for β-HCG testing. Ensure that the test is negative before proceeding; a positive test will exclude the volunteer from the study.
4. Record vital signs (blood pressure, temperature, heart rate, and respiratory rate).

5. Administer the vaccine.
6. Observe for at least 30 minutes after vaccination and evaluate for immediate adverse reactions.
7. Education by study staff during the 30 minute post-immunization waiting period describing proper use of digital thermometers, the proper method of filling out diary card, signs and symptoms of potential adverse events, and how and when to contact study staff.

Study Day 182 (Day 2 post second vaccination)
1. Perform interim history and focused physical exam, emphasizing examination of any acute complaints.
2. Record vital signs.
3. Obtain approximately 15 mL of blood for CBC with differential and virology.

Study Day 184 (Day 4 post second vaccination ± 1 day)
1. Perform interim history and focused physical exam, emphasizing examination of any acute complaints.
2. Record vital signs.
3. Obtain approximately 25 mL of blood for CBC with differential, PT/PTT, ALT, and virology.

Study Day 186 (Day 6 post second vaccination ± 1 day)
1. Perform interim history and focused physical exam, emphasizing examination of any acute complaints.
2. Record vital signs.
3. Obtain approximately 20 mL of blood for CBC with differential, ALT, and virology.

Study Day 188 (Day 8 post second vaccination ± 1 day)
1. Perform interim history and focused physical exam, emphasizing examination of any acute complaints.
2. Record vital signs.
3. Obtain approximately 35 mL of blood for CBC with differential, PT/PTT, ALT, virology, and immunology (humoral and cellular immune studies).

Study Day 190 (Day 10 post second vaccination ± 1 day)
1. Perform interim history and focused physical exam, emphasizing examination of any acute complaints.
2. Record vital signs.
3. Obtain approximately 20 mL of blood for CBC with differential, ALT, and virology.

Study Day 192 (Day 12 post second vaccination ± 1 day)
1. Perform interim history and focused physical exam, emphasizing examination of any acute complaints.
2. Record vital signs.

3. Obtain approximately 25 mL of blood for CBC with differential, PT/PTT, ALT, and virology.

Study Day 194 (Day 14 post second vaccination ± 1 day)

1. Perform interim history and focused physical exam, emphasizing examination of any acute complaints.
2. Record vital signs.
3. Obtain approximately 40 mL of blood for CBC with differential, ALT, virology, and immunology (humoral and cellular immune studies).

Study Day 196 (Day 16 post second vaccination ± 1 day)

1. Perform interim history and focused physical exam, emphasizing examination of any acute complaints.
2. Record vital signs.
3. Obtain approximately 25 mL of blood for CBC with differential, PT/PTT, ALT, and virology.

Study Day 201 (Day 21 post second vaccination ± 1 day)

1. Perform interim history and focused physical exam, emphasizing examination of any acute complaints.
2. Record vital signs.
3. Obtain approximately 15 mL of blood for ALT and immunology (antibody and cellular immune studies). Hematology will be done if day 196 WBC, ANC, platelet count, or hemoglobin results are outside of the normal laboratory ranges. Virology will be done on volunteers who had vaccine virus recovered from the blood on Study Day 196 and will be repeated at each visit until viremia has cleared.
4. Collect diary card.

Study Day 208 (Day 28 post second vaccination ± 2 days)

1. Perform interim history of complaints, including receipt of treatment that could interfere with immune assessment.
2. Record vital signs.
3. Obtain approximately 25 mL of blood for immunology (antibody and cellular immune studies) and PT/PTT. ALT will be done if indicated (if values from Study Day 208 were abnormal and clinically significant).

Study Day 222 (Day 42 post second vaccination ± 2 days)

1. Perform interim history of complaints, including receipt of treatment that could interfere with immune assessment
2. Record vital signs.
3. Obtain approximately 20 mL of blood for immunology (antibody and other immunological assays).

7.5 Volunteer Temperature Diary

Volunteers will be asked to record their temperature three times each day (for Study Days 0-16 post-each vaccination) on a diary card provided on the day of vaccination (included in supplements to the protocol**)**. Volunteers will be given a digital oral thermometer to take their temperatures. Study staff will instruct volunteers how to take their temperature and to take their temperature at approximately the same time each day as well as to take additional temperatures if they feel they have an elevated temperature. Volunteers will be instructed to wait at least 15 minutes after eating, drinking, or smoking to take their temperature. They will be asked to confirm an elevated temperature (≥100. 4 °F) by retaking the temperature after a 20 minute interval. A fever will be defined as an oral temperature of ≥100.4°F from two consecutive readings ≥1 hour apart.

7.6 Clinical Laboratory Testing

Using standard techniques, University of Maryland Laboratories, Quest Laboratories (for the Washington DC site), or another Clinical Laboratory Improvement Amendments (CLIA) certified laboratory will perform the following tests:

1. Complete blood count plus white blood cell differential
2. PT/PTT
3. Serum creatinine
4. CPK
5. AST, ALT, Alkaline phosphatase, total bilirubin
6. HIV assay (screening antibody assay with Western blot confirmation).
7. HBsAg ELISA
8. HCV (HCV screening antibody assay with immunoblot confirmation or viral PCR confirmation).
9. Urinalysis (in the event of an abnormal urine dipstick test)

Urine β-HCG testing will be performed at the clinical study site using an FDA-approved urine pregnancy test kit. Urine dipstick testing will be performed at the study site using an FDA-approved product. Determination of rDEN1Δ30 virus titer and antibody assays to dengue and other flaviviruses will be done at the clinical study site laboratory.

7.7 Virologic Assays

Vaccine virus will be titered by standard plaque assay using laboratory-established protocols. Briefly, plasma or serum is diluted by serial 10-fold dilution in media and plaqued in duplicate onto Vero cells. Virus is adsorbed for 1 hour at 37°C and then overlaid with 1% methylcellulose and assayed by immunostaining after incubation for 5 days at 37°C. In addition, virus is amplified by inoculating 0.3 mL of plasma or serum onto Vero cells directly, overlaying with 3 mL of media, incubating at 37°C for 5 days and then titering as above. The methods outlined above may change per the established laboratory protocol. Vaccine virus recovered from the serum of volunteers will be sequenced around the Δ30 region of the virus to evaluate the stability of the Δ30 mutation

7.8 Immunology Testing

7.8.1 Antibody Assay

Antibody levels to DEN1 will be measured by serum plaque reduction neutralizing antibody assay per standard laboratory protocols.

7.8.2 Other Immunological Assays

PBMCs may be collected for several immunological assays including, but not limited to, phenotyping of DEN virus-infected PBMCs, epitope mapping, cytokine analysis, memory function, and T-cell stimulation assays. Cytokine levels in serum may also be measured.

7.9 Skin Biopsy

We will perform an **optional** skin biopsy sub-study in order to assess the underlying mechanisms related to the development of skin rash seen in prior dengue vaccine studies and in order to determine cellular targets for dengue virus infection.

A prior skin biopsy study was performed in patients with DHF and skin rash. Immunofluorescent staining of the skin specimens showed deposits of IgM, β_1C-globulin, dengue antigen, and fibrinogen. These findings suggested that the cutaneous rashes occurring in DHF were caused by an immunopathologic process; however, the studies performed were limited and not conclusive.

In the initial Phase 1 and Phase 2 studies of the dengue 4 vaccine candidate virus, DEN4Δ30, approximately 50% of all volunteers, regardless of dose received, developed a maculopapular rash. This rash was typically seen between Day 10 and Day 14 of follow-up and was localized to the upper and lower extremities and truncal area. It is unclear whether the underlying pathogenesis of this rash was immune-mediated or directly related to the dengue 4-based virus.

Because the rDEN1Δ30 vaccine is a live attenuated dengue virus and DEN1 is known to cause rash in wild-type infections, it is expected that we may observe a similar rash in rDEN1Δ30 vaccinees. Therefore, we plan to perform a sub-study in which we will perform skin biopsies. This is an **optional** sub-study which will be offered to all individuals who enter the parent study, except for those individuals who have a history of keloid development after skin puncture procedures (i.e., ear piercing). Whether or not an individual wishes to enroll in the sub-study will not affect in any way their eligibility to enroll in the parent study.

The biopsy will be performed using a standard skin punch technique [23]. In individuals who develop a rash, two (3 mm) full thickness skin biopsies will be collected; one from the rash site and one from another site that is clear of rash. Those volunteers who do not develop a rash (control volunteers) will only have one skin biopsy performed at an appropriate control site to be determined at the time of examination just prior to skin biopsy. Skin biopsy specimens will be collected in a sterile container. Each specimen will be sectioned and placed in either preservative

or in culture media. Skin specimens will then be studied to identify immune complexes, complement, IgM and IgG, rDEN1Δ30 virus, DEN1 antibody, and other immune mediators.

8.0 ADVERSE EVENT MONITORING

8.1 Definitions

8.1.1 Adverse Event

An adverse event (AE) is any untoward medical occurrence in a study participant administered the vaccine or placebo and does not necessarily have a causal relationship with the vaccination. An AE can therefore be any unfavorable and unintended sign (including an abnormal laboratory finding), symptom, or disease temporally associated with the use of the investigational vaccine whether or not related to it. This includes exacerbation of pre-existing conditions and intercurrent illnesses. All AEs must be graded for intensity and relationship to the investigational vaccine as described in **Sections 8.2.2** and **8.2.3** in this protocol. All adverse events experienced by a volunteer will be recorded in the Adverse Event log in the volunteer chart. Adverse events will be categorized as local reactogenicity, solicited reactogenicity, or other adverse event.

8.1.2 Serious Adverse Event

A serious adverse event (SAE) is an AE, whether considered related to the investigational vaccine or not, meeting one of the following conditions:

1. Death during the period of protocol-defined surveillance.
2. Life threatening: defined as an event that places a subject at immediate risk of death at the time of the event and does not refer to an event that hypothetically might have caused death were it more severe.
3. Hospitalization during the period of protocol-defined surveillance: defined as at least an overnight stay in the hospital or emergency ward for treatment that would have been inappropriate if administered in the outpatient setting.
4. Results in a congenital anomaly or birth defect.
5. Results in a persistent or significant disability or incapacity; defined as a substantial disruption of the study participant's ability to carry out normal life functions.

Any other important medical event that may not result in death, be life threatening, or require hospitalization may be considered a serious AE when, based upon appropriate medical judgment, the event may jeopardize the subject and may require medical or surgical intervention to prevent one of the outcomes listed above.

8.2 Assessment of Adverse Events

8.2.1 Identification of Adverse Events

Assessment of safety will include clinical observations and monitoring of hematological, blood chemistry, and immunologic parameters. Safety will be evaluated by monitoring of the volunteers for local and systemic adverse reactions during the course of the study from the administration of the vaccine through the last follow-up visit. Solicited adverse events will be collected through study day 28. All Serious Adverse Events will be collected for the duration of the study. Volunteers will be closely monitored for 30 minutes following each immunization. Additionally, volunteers will return to the clinic on Study Days 2, 4, 6, 8, 10, 12, 14, 16, and 21 post-vaccination. It is during this time period that we anticipate AEs related to infection with the vaccine virus will be manifested, hence the volunteers will be seen frequently. Volunteers will be asked to monitor and record their oral temperature 3 times a day for Study Days 0 – 16 (post each vaccination). At each visit, they will be queried about possible vaccine-related AEs (solicited adverse events) and have a focused physical exam performed. All AEs will be graded for intensity and relationship to study product. Reactions will be graded as described in **Section 8.2.2** of this protocol. A study clinician will be available by telephone or pager 24 hours a day during the study evaluation period.

8.2.2 Determination of Severity

All AEs will be assessed by the investigator using the protocol-defined adverse event severity scale as described in **Table 5**.

Table 5: AE Grading Table

Severity	Definition
Grade 0 (None)	None
Grade 1 (Mild)	Event that is easily tolerated.
Grade 2 (Moderate)	Event that interferes with daily activity, or treatment is given
Grade 3 (Severe)	Event that prevents daily activity.

Intensity of local reactogenicity and solicited reactogenicity will be assessed by the investigator as described in **Table 6**. Selected laboratory AEs will be graded for severity as defined in **Table 7**. All other clinically significant laboratory AEs and clinical AEs will be graded for severity following the toxicity table in **Appendix 1**.

Table 6: Assessment of Adverse Event Intensity

Local Reactogenicity	Grade	Intensity

Pain at injection site	0<br>1<br>2<br>3	Absent<br>Pain that is easily tolerated<br>Pain that interferes with daily activity<br>Pain that prevents daily activity
Erythema at injection site	0<br>1<br>2<br>3	0 mm<br>>0 - ≤20 mm<br>>20 - ≤50 mm<br>>50 mm
Swelling at injection site	0<br>1<br>2<br>3	0 mm<br>>0 - ≤20 mm<br>>20 - ≤50 mm<br>>50 mm
Pruritus at injection site	0<br>1<br>2<br>3	Absent<br>Pruritus that is easily tolerated<br>Pruritus that interferes with daily activity<br>Pruritus that prevents daily activity
**Solicited Reactogenicity**	**Grade**	**Intensity**
Fever (oral).<br>Must be recorded on two consecutive measurements ≥1 hour apart.	0<br>1<br>2<br>3	<100.4 F (<38°C)<br>100.4° F – 101.4°F (38.0°C – 38.6°C)<br>101.5°F – 102.4°F (38.7°C – 39.1°C)<br>>102.4°F (>39.1°C)
Headache	0<br>1<br>2<br>3	None<br>Headache that is easily tolerated<br>Headache that interferes with daily activity<br>Headache that prevents daily activity
Retro-orbital pain	0<br>1<br>2<br>3	None<br>Retro-orbital pain that is easily tolerated<br>Retro-orbital pain that interferes with daily activity<br>Retro-orbital pain that prevents daily activity
Nausea	0<br>1<br>2<br>3	None<br>Nausea that is easily tolerated<br>Nausea that interferes with daily activity<br>Nausea that prevents daily activity
Photophobia (eye pain)	0<br>1<br>2<br>3	None<br>Photophobia that is easily tolerated<br>Photophobia that interferes with daily activity<br>Photophobia that prevents daily activity
Malaise	0<br>1<br>2<br>3	None<br>Malaise that is easily tolerated<br>Malaise that interferes with daily activity<br>Malaise that prevents daily activity
**Solicited Reactogenicity**	**Grade**	**Intensity**
Myalgia	0<br>1<br>2<br>3	None<br>Myalgia that is easily tolerated<br>Myalgia that interferes with daily activity<br>Myalgia that prevents daily activity
Arthralgia	0<br>1<br>2<br>3	None<br>Arthralgia that is easily tolerated<br>Arthralgia that interferes with daily activity<br>Arthralgia that prevents daily activity

Rash	0<br>1<br>2<br><br>3	None<br>Rash is present but asymptomatic<br>Rash is symptomatic (pruritus/pain) but does not interfere with function<br>Rash is symptomatic and interferes with function

Table 7: Selected Laboratory Toxicity Table

HEMATOLOGY	Grade 1	Grade 2	Grade 3	Grade 4
Hemoglobin (Decrease from testing laboratory LLN in gm/dL)	9.5 g/dL–10.5 g/dL **OR** any decrease ≥2.5 g/dL	8.0 g/dL –9.4 g/dL **OR** any decrease ≥3.5 g/dL	6.5 g/dL–7.9 g/dL **OR** any decrease ≥4.5 g/dL	<6.5 g/dL
Absolute neutrophil count (ANC)	1000-1500/mm^3	750-999/mm^3	500- 749/mm^3	<500/mm^3
Platelets	120,000 – 100,000/mm^3	99,999 – 75,000/mm^3	74,999– 50,000/mm^3	<50,000/mm^3
PT	>1.0-1.24 x ULN	1.25-1.49 x ULN	1.5-3.0 x ULN	>3.0x ULN
PTT	>1.0-1.66 x ULN	>1.66-2.33 x ULN	>2.33-3.0 x ULN	>3.0 x ULN
**CHEMISTRY**				
ALT	1.25 – 3.0 x ULN	>3.0 – 5.0 x ULN	>5.0 – 10 x ULN	>10 x ULN

8.2.3 Association with Receipt of Study Vaccine

All AEs will have their relationship to study vaccine assessed using the following terms:

Definitely: Clear-cut temporal association, and no other possible cause.
Probably: Clear-cut temporal association and a potential alternative etiology is not apparent.
Possibly: Less clear temporal association; other etiologies also possible.
Unlikely: Temporal association between the AE and the vaccine or the nature of the event is such that the vaccine is not likely to have had any reasonable association with the observed illness/event (cause and effect relationship improbable but not impossible).
Not related: The AE is completely independent of vaccine administration; and/or evidence exists that the event is definitely related to another etiology.

The degree of certainty with which an AE can be attributed to administration of the study vaccine will be determined by how well the event can be understood in terms of one or more of the following:

1. The event being temporally related with vaccination or reproduced on re-vaccination.
2. A reaction of similar nature having previously been observed with this type of vaccine and/or formulation.

3. The event having often been reported in the literature for similar types of vaccines.

All local injection-site reactions will be considered causally related to vaccination.

8.3 Adverse Event Reporting

All SAEs will be reviewed by a study physician, recorded on the appropriate SAE form, and followed through to resolution or until the study physician deems the event to be chronic or the subject to be stable. All SAEs will be reported by telephone or fax within 1 working day of notification of the SAE occurrence to all of the following:

- Sponsor: Regulatory Compliance and Human Subjects Protection Branch (RCHSPB): Phone 301-846-5301, Fax: 301-846-6224, rchspsafety@mail.nih.gov
- Western IRB Phone: 800-562-4789, Fax: 360-252-2498: will be notified according to WIRB guidelines.

All SAEs determined to be possibly, probably, or definitely related to vaccine will be reported to:

- The DSMB executive secretary: Phone 301-846-5301, Fax: 301-846-6224
- The Institutional Biosafety Committee: Phone 410-955-5918, Fax 410-955-5929

Following notification from the investigator, RCHSPB as the IND Sponsor, will report events that are both serious and unexpected that are unlikely, possibly, probably, or definitely related to the vaccine, to the FDA within the required timelines: fatal and life-threatening events within 7 calendar days (by phone or fax) and all other SAEs in writing within 15 calendar days. All SAEs not listed as unlikely, possibly, probably, or definitely related or are expected events will be reported to the FDA at least annually in a summary format. All local and systemic reactions not meeting the criteria for "serious adverse events" will be captured on the appropriate case report form (CRF). These events will be followed to resolution or until a study physician deems the event to be chronic or the subject to be stable. This decision will be documented in the study record.

8.4 Adverse Event Monitoring

8.4.1 NIAID Intramural Data and Safety Monitoring Board

The NIAID Intramural DSMB is constituted to review the safety data of Intramural NIAID clinical studies that require DSMB oversight and consists of experts in infectious diseases, biostatistics, and clinical studies. The Principal Investigator will also provide the DSMB Secretary with blinding codes in a sealed envelope in case the DSMB requires this information to make its recommendations. The DSMB is responsible for reviewing the IRB approved protocol, informed consent documents, the data and safety monitoring plan, and the stopping guidelines prior to initiation of the study,

unless otherwise waived by the NIAID Clinical Director. At their discretion, the DSMB may recommend modification of the protocol.

The DSMB will review cumulative study data twice per year to assess the performance of overall study operations to evaluate safety, study conduct, and scientific validity and integrity of the trial and any other relevant issues, as necessary, per the official DSMB policy.

If a dose of vaccine is considered unacceptably reactogenic, additional vaccinations will be suspended until reviewed with the DSMB and study Sponsor (RCHSPB).

The following criteria will be used to define unacceptable reactogenicity of the rDEN1Δ30 vaccine:

1. One or more volunteers experience a Serious Adverse Event (as defined in **Section 8.1.2** in this protocol) that is determined to be possibly, probably, or definitely related to the vaccine (as defined in **Section 8.2.3** in this protocol), **OR**
2. One or more volunteers experience anaphylaxis that is probably or definitely related to the vaccine **OR**
3. Any severe clinical illness occurs that is not explained by a diagnosis that is unrelated to vaccination, **OR**
4. One or more volunteers in a single dose cohort experience an objective physical finding of Grade 3 or higher that is definitely, probably, or possibly related to vaccine (with the exception of isolated Grade 3 erythema at the injection site), as defined in **Section 8.2.2.** in this protocol, **OR**
5. One or more volunteers in a dose cohort experience a Grade 3 or higher elevation in serum ALT, **OR**
6. One or more volunteers in a single dose cohort experience a Grade 3 or higher prolongation of PT or PTT, **OR**
7. One or more volunteers in a single dose cohort experience a Grade 3 or higher degree of thrombocytopenia, **OR**
8. One or more volunteers in a single dose cohort experience a dengue-like syndrome defined as: **Infection[1] associated with fever and two or more of the following symptoms**:

    a. Grade 2 headache lasting ≥12 hours
    b. Grade 2 photophobia lasting ≥12 hours
    c. Generalized myalgia, Grade 2, lasting ≥12 hours
    d. Retro-orbital pain, Grade 2, lasting ≥12 hours
    e. Sustained or intermittent epistaxis for >24 hours

[1] Infection is defined as recovery of vaccine virus from the blood or serum of a volunteer and/or a ≥4-fold rise in serum neutralizing antibody against DEN1.

9.0 DATA COLLECTION AND MONITORING

9.1 Source Documentation

Complete source documentation (laboratory test reports, hospital or medical records, etc.) is required for every study volunteer for the entire duration of the study. Source documentation and volunteer symptom diaries will be used to record data for volunteers enrolled in the study onto case report forms (CRFs). The Principal Investigator is responsible for the accuracy and completeness of the data reported to the Sponsor in the CRFs. Data reported on the CRFs derived from source documents should be consistent with source documents or the discrepancies should be explained. Source documentation will be made available for review or audit by the Sponsor or designee and any applicable Federal authorities.

9.2 Study Documentation

Study-related documentation will be completed as required by the IRBs, the Sponsor, and regulatory authorities. Continuing review documentation will be submitted by the Investigator to the IRBs on the anniversary date of initial review as specified by each IRB. An annual report will be submitted by the Sponsor to the FDA based on the date that the IND for rDEN1Δ30 chimeric dengue vaccine went into effect. These reports will provide a brief description of the progress of the investigation as outlined in 21*Code of Federal Regulations* 312.33, and will include descriptions of any revisions of the protocol.

The Principal Investigator will maintain adequate records of the disposition of the investigational product, including dates of receipt and disposition, quantity, and use by volunteers. If the study is terminated, suspended, or completed, the Principal Investigator will return all unused supplies of the investigational product to the current NIAID-contracted repository.

9.3 Retention of Specimens

All specimens collected as part of this study may, with the volunteer's permission, be stored for future research as part of our approved biosample repository for vaccine research. These samples may be used to learn more about flavivirus infection and other diseases. These samples will not be sold or used to make commercial products. Genetic tests will not be performed on these samples. Samples will be stored only with the volunteer's permission. All samples stored in the repository will be labeled with the study identification (ID) numbers of the participants that, by themselves, cannot identify study volunteers but are linkable to other research databases (e.g., from questionnaires, clinical assessments, logbooks, etc.) generated by the main study. The repository database will contain only the study participant ID numbers. A master log linking the study participant ID numbers to the name of the participant will be maintained in a password-protected database system with access limited to authorized research team members.

9.4 Retention of Records

Study-related documents will be maintained by the Principal Investigator for a period of 2 years after final marketing approval of the vaccine, or if 2 years have elapsed since the formal discontinuation of clinical development of the product. The Sponsor is required to inform the Principal Investigator as to when such documents need no longer be retained. Storage of all study-related documents will be such that confidentiality will be strictly maintained. Should the investigator wish to assign the study records to another party the investigator must provide written notification of such intent to RCHSPB/NIAID with the name of the person who will accept responsibility for the transferred records and/or their new location.

9.5 Protocol Revisions

No revisions to this protocol will be permitted without documented approval from both the Sponsor and the IRBs that granted the original approval for the study. This does not apply to changes made to reduce discomfort or avert risk to study volunteers. Furthermore, in the event of a medical emergency, the Principal Investigator shall perform any medical procedures that are deemed medically appropriate. The Principal Investigator must notify the Sponsor of all such occurrences.

9.6 Clinical Investigator's Brochure

Investigators will receive the current version of the Clinical Investigator's Brochure, which comprehensively describes all the available preclinical experience with the experimental vaccine. If relevant new information becomes available during the course of the study, all study Investigators will receive a revised Investigator's Brochure or an amendment to the current version.

9.7 Study Monitoring

The Sponsor will monitor all aspects of the study with respect to current Good Clinical Practices and for compliance with applicable government regulations. Prior to the start of the study, the Principal Investigator will be informed of the frequency of monitoring visits and will be given reasonable notification prior to each visit. The objectives of a monitoring visit will be to verify the prompt reporting of SAEs, to check the availability of the signed Informed Consent, and to compare CRFs and spreadsheets with source data for completeness and accuracy. During the monitoring visit, the Principal Investigator (and/or designee) and other study personnel will be available to discuss the study. Study documents must be available for review throughout the course of the study. The Sponsor will retain originals of the FDA Form 1572 and copies of other study documents as deemed necessary.

10.0 STATISTICAL CONSIDERATIONS

10.1 General Design

The goal of this Phase 1 vaccine study is to demonstrate the safety and immunogenicity of the rDEN1Δ30 dengue vaccine candidate in human volunteers. The purpose of this Phase 1 study is to evaluate a DEN1 vaccine candidate for dose as measured by low reactogenicity, immune response, and persistence of antibody.

10.1.1 Description of the Statistical Methods to be Employed

This study, like other Phase 1 studies, is basically exploratory rather than confirmatory; its purpose is to estimate adverse event rates and patterns of immune responses rather than to test formal statistical hypotheses. Descriptive approaches will be used to meet the protocol objectives as stated in **Section 3.0** of this protocol, as well as formal statistical tests as outlined below. Results will be presented in tabular format, as well as graphically where appropriate.

<u>Primary Objective 1</u>: To determine safety and immunogenicity of the rDEN1Δ30 vaccine given as two doses separated by 4 or 6 months:

- Summarize the frequency of immediate, systemic, and local AEs following each vaccination in cohort 1 and cohort 2.
- Line listing of individual clinical and laboratory AEs classified as systemic, local, and other will be displayed in tabular format and stratified by each dose in cohort 1 and cohort 2.
- AEs will be summarized by severity and relationship to vaccine by individuals, each dose, and each cohort.

<u>Primary Objective 2</u>: To determine the optimum interval between first and second (booster) dose of the rDEN1Δ30 candidate vaccine as assessed by the neutralizing antibody response to DEN1induced by the vaccine at 4 and 6 weeks after first and second vaccination. The Tukey Kramer multiple comparison post test will be used to compare the means of the different cohorts at Study Days 28 and 42 post-second vaccination.

<u>Secondary Objective 1</u>: To assess the frequency, magnitude, and duration of viremia following each vaccination. The mean peak viremia, mean day of onset of viremia, and mean duration of viremia of each dose cohort will be calculated. The Tukey Kramer multiple comparison post test will be used to determine significant differences between groups within cohorts and between cohorts.

<u>Secondary Objective 2</u>: To determine the number of vaccinees infected with rDEN1Δ30. Infection is defined as recovery of vaccine virus from

the blood or serum of a volunteer and/or by seroconversion to DEN1 (a ≥4-fold rise in DEN1 neutralizing antibodies). The Tukey Kramer multiple comparison post test will be used to determine significant differences between groups.

Secondary Objective 3: If both doses of vaccine are administered, to compare the infectivity rates, safety, and immunogenicity between cohorts. The Tukey Kramer multiple comparison post test will be used to determine significant differences between groups.

Secondary Objective 4: To evaluate the immunopathological mechanism of vaccine-associated rash in those volunteers who are willing to undergo skin biopsy. The analysis will be primarily descriptive.

Secondary Objective 5: To evaluate the phenotype and activation of peripheral blood mononuclear cells (PBMCs) at primary infection and challenge with DEN1. The analysis will be primarily descriptive

10.1.2 Safety

The primary safety endpoint is the frequency of vaccine-related AEs, as classified by both intensity and severity through active and passive surveillance. Separate assessments of systemic and local reactions will be performed.

10.1.3 Immunogenicity

Anti-rDEN1Δ30 neutralizing antibody will be measured on Study Days 0, 28, and 42 following each vaccination for cohort 1 and cohort 2. Seroconversion will be defined as a ≥4-fold rise in serum neutralizing antibody titer compared with Study Day 0. Anti-rDEN1Δ30 neutralizing antibody will be measured on Study Days 120, 148, and 162 for cohort 1. Anti-rDEN1Δ30 neutralizing antibody will be measured on Study Days 180, 208, and 222 for cohort 2.

11.0 PROTECTION OF HUMAN SUBJECTS

11.1 Institutional Review Board

The Principal Investigator will be responsible for obtaining IRB approval for the study. Before the start of the study, the appropriate documents (including the Protocol, Investigator's Brochure, Informed Consent Form, information sheets, and advertisements) will be submitted to, and approved by, the Western Institutional Review Board. A copy of the study approval (including approval of the informed consent form) is to be maintained in the Principal Investigator's study document binder and a copy will be supplied to the Sponsor. During the study, the Principal Investigator is responsible for providing the IRB with all documents to review (i.e., Protocol Amendments, informed consent form updates, advertisements, and any

written information that may be provided to the volunteer). Annual reports on the progress of the study will be made to the IRB by the Principal Investigator in accordance with IRB guidelines and government regulations.

11.2 Informed Consent

In obtaining and documenting informed consent, the Principal Investigator must comply with the applicable regulatory requirements, GCP, and ethical principles. The written informed consent form must be approved by the IRB prior to its use. The participants may withdraw consent at any time throughout the course of the study. A copy of the informed consent document will be given to the participants for their records. The rights and welfare of the participants will be protected by emphasizing to them that the quality of their medical care will not be adversely affected if they decline to participate in this study.

11.3 Risks

Risks to the volunteers are associated with venipuncture, skin biopsy, and with immunization. These risks are outlined below. Female participants will be cautioned of the unknown risk of study vaccines to the fetus and will be advised to use effective birth control methods for the duration of the study.

11.4 Venipuncture

The total amount of blood to be drawn throughout the duration of the study, including screening, is approximately 700 mL. Risks occasionally associated with venipuncture include pain and bruising at the site of venipuncture, lightheadedness, and syncope (rarely)..

11.5 Skin Biopsy

The potential risks of a skin biopsy include risk of infection, temporary pain, and risk of redness and scarring at the biopsy site. The risk of infection is quite low, occurring in fewer than 1 in 200 individuals after the procedure (personal communication, J. C. McArthur, Director of Cutaneous Nerve Laboratory [24]). We will perform the skin biopsy using aseptic technique. Sutures will not be used. The risk of temporary pain with the injection of local anesthetic is transient only. There usually is a burning sensation that lasts approximately 5-10 seconds after injection. The risk of redness in the healing biopsy is also transient. This redness usually fades as the biopsy heals over 2-3 weeks. The potential for severe scarring exists but is also very low. Other studies have shown that fewer than 1 in 500 individuals develop permanent scarring. We will not perform skin biopsies on individuals who have a history of keloid development post skin puncture procedures (i.e., ear piercing). Each volunteer will be informed of these potential risks in detail.

11.6 Immunization

Possible local vaccine reactions include pain, swelling, or erythema for 2 to 3 days, lymphadenopathy, or pruritus at the injection site. Systemic reactions such as rash and transient neutropenia have been observed in some volunteers infected with the dengue 1 vaccine candidate virus, rDEN1Δ30 [25]. Other potential systemic reactions

that may occur include symptoms of dengue such as fever, headache, eye pain, photophobia, generalized myalgias, or decreased platelet count. Immediate hypersensitivity reactions including urticaria, anaphylaxis, or other IgE-mediated responses are possible as with any vaccine. Volunteers who receive the rDEN1Δ30 vaccine may be at increased risk of more severe disease (DHF/DSS) should they become infected with a different DEN virus serotype in the future.

As with any investigational vaccine, there is a theoretical possibility of risks about which we have no present knowledge. Volunteers will be informed of any such risks should further information become available.

11.7 Benefits

Volunteers will not receive any direct benefit from participation in this study. It is hoped that information gained in this study will contribute to the development of a safe and effective vaccine for the prevention of Dengue 1.

11.8 Compensation

Volunteers will be paid $75 for screening, $100 for each vaccination, and $75 for each completed scheduled follow-up visit. They will also receive a $250 bonus if all study visits are completed on time (total = $2250). Volunteers will only be paid for the screening if they are ***enrolled*** in the study. Volunteers will only be paid for the visits that they complete.

11.9 Confidentiality

All study-related information will be stored securely at the study site. All participant information will be stored in locked file cabinets in areas with access limited to study staff. All laboratory specimens, reports, study data collection, process and administrative forms will be identified by coded number only to maintain participant confidentiality. Computer entry will be done by coded patient identification numbers, and all local databases will be secured with password-protected access systems. Forms, lists, logbooks, appointments books, and any other listings that link participant ID numbers to other identifying information will be stored in a separate, locked file in an area with limited access. Participants' study information will not be released without the written permission of the participant, except as necessary for monitoring by the Sponsor and/or its contractors and the FDA.

11.10 Biohazard Containment

As the transmission of HIV and other blood-borne pathogens can occur through contact with contaminated needles, blood, and blood products, appropriate blood and secretion precautions will be employed by all personnel in the drawing of blood and shipping and handling of all specimens for this study, as currently recommended by the Centers for Disease Control and Prevention and the National Institutes of Health.

All infectious specimens will be transported using packaging mandated in the Code of Federal Regulations, 42 CFR Part 72. Please also refer to individual carrier guidelines, e.g., Federal Express, Airborne Express, for specific instructions.

12.0 PUBLICATION POLICY

The International Committee of Medical Journal Editors (ICMJE) member journals have adopted a trials-registration policy as a condition for publication. This policy requires that all clinical trials be registered prior to enrollment of any subject in a public trials registry such as ClinicalTrials.gov, which is sponsored by the National Library of Medicine. The ICMJE defines a clinical trial as any research project that prospectively assigns human participants to intervention or comparison groups to study the cause-and-effect relationship between a medical intervention and a health outcome. Studies designed for other purposes, such as to study pharmacokinetics or major toxicity, would be exempt from this policy.

13.0 REFERENCES

1. Monath T, Heinz F. Flaviviruses. In: Fields BN, Knipe DM, Howley PM, et al., eds. Fields Virology. Third ed., Vol. 1. Philadelphia: Lippincott-Raven, 1996:961-1034

2. WHO. Dengue haemorrhagic fever: diagnosis, treatment, prevention and control. 2nd ed. Geneva: WHO, 1997

3. Sabin A. Recent advances in our knowledge of Dengue and Sandfly Fever. Amer J Trop Med Hyg. 1955;4:198-207

4. Burke DS, Nisalak A, Johnson DE, and Scott RM. A prospective study of dengue infections in Bangkok. Am J Trop Med Hyg. 1988;38:172-80

5. Wu SJ, Grouard-Vogel G, Sun W, et al. Human skin Langerhans cells are targets of dengue virus infection. Nat Med. 2000;6(7):816-20

6. Couvelard A, Marianneau P, Bedel C, et al. Report of a fatal case of dengue infection with hepatitis: demonstration of dengue antigens in hepatocytes and liver apoptosis. Hum Pathol. 1999;30:1106-10

7. Kuo CH, Tai DI, Chang-Chien CS, Lan CK, Chiou SS, and Liaw YF. Liver biochemical tests and dengue fever. Am J Trop Med Hyg. 1992;47:265-70

8. Marianneau P, Steffan AM, Royer C, et al. Infection of primary cultures of human Kupffer cells by Dengue virus: no viral progeny synthesis, but cytokine production is evident. J Virol. 1999;73:5201-6

9. Whitehead SS, Hanley KA, Blaney JE, Jr., Gilmore LE, Elkins WR, and Murphy BR. Substitution of the structural genes of dengue virus type 4 with those of type 2 results in chimeric vaccine candidates which are attenuated for mosquitoes, mice, and rhesus monkeys. Vaccine. 2003; Oct 1;21(27-30):4307-16

10. Sabin AB, Schlesinger RW. Production of immunity to dengue with virus modified by propagation in mice. Science. 1945;101:640-642

11. Schlesinger RW, Gordon I, Frankel JW, Winter JW, Patterson PR, and Dorrance WR. Clinical and serologic response of man to immunization with attenuated dengue and yellow fever viruses. J Immunol. 1956;77:352-64

12. Wisseman CJ, Sweet B, Rosenzweig E, and Eylar O. Attenuated living type 1 dengue vaccines. Am J Trop Med Hyg. 1963;12:620-623

13. Bellanti JA, Bourke A, Buescher E, et al. Report of Dengue Vaccine Field Trial in the Caribbean, 1963: A Collaborative Study. Abreges des Communications:93

14. Edelman R, Tacket CO, Wasserman SS, et al. A live attenuated dengue-1 vaccine candidate (45AZ5) passaged in primary dog kidney cell culture is attenuated and immunogenic for humans. J Infect Dis. 1994;170:1448-55

15. Edelman R, Wasserman SS, Bodison SA, et al. Phase 1 trial of 16 formulations of a tetravalent live-attenuated dengue vaccine. Am J Trop Med Hyg. 2003;69:48-60

16. Kanesa-Thasan N, Sun W, Kim-Ahn G, et al. Safety and immunogenicity of attenuated dengue virus vaccines (Aventis Pasteur) in human volunteers. Vaccine. 2001;19:3179-88

17. Sun W, Edelman R, Kanesa-Thasan N, et al. Vaccination of human volunteers with monovalent and tetravalent live-attenuated dengue vaccine candidates. Am J Trop Med Hyg. 2003;69:24-31

18. Bhamarapravati N, Sutee Y. Live attenuated tetravalent dengue vaccine. Vaccine 2000;18:44-47

19. Sabchareon A, Lang J, Chanthavanich P, et al. Safety and immunogenicity of tetravalent live-attenuated dengue vaccines in Thai adult volunteers: role of serotype concentration, ratio, and multiple doses. Am J Trop Med Hyg. 2002;66:264-72

20. Whitehead SS, Falgout B, Hanley KA, Blaney Jr JE, Jr., Markoff L, and Murphy BR. A live, attenuated dengue virus type 1 vaccine candidate with a 30-nucleotide deletion in the 3' untranslated region is highly attenuated and immunogenic in monkeys. J Virol. 2003;77:1653-7

21. Blaney JE, Jr., Durbin AP, Murphy BR, and Whitehead SS. Development of a live attenuated dengue virus vaccine using reverse genetics. Viral Immunol. 2006;19(1):10-32

22. Durbin AP, Karron RA, Sun W, et al. Attenuation and immunogenicity in humans of a live dengue virus type-4 vaccine candidate with a 30 nucleotide deletion in its 3'-untranslated region. Am J Trop Med Hyg. 2001;65:405-13

23. McCarthy BG, Hsieh ST, Stocks A, et al. Cutaneous innervation in sensory neuropathies: evaluation by skin biopsy. Neurology. 1995;45:1848-55

24. McArthur JC, Stocks EA, Hauer P, Cornblath DR, and Griffin JW. Epidermal nerve fiber density: normative reference range and diagnostic efficiency. Arch Neurol. 1998;55:1513-20

25. Durbin AP, McArthur J, Marron JA, et al. The live attenuated dengue serotype 1 vaccine rDEN1Delta30 is safe and highly immunogenic in healthy adult volunteers. Hum Vaccin. 2006;Jul-Aug;2(4):167-73

14.0 Appendix 1: Toxicity Table for Grading Adverse Events, Adapted from the HIV Vaccine Trials Network

Parameter	Grade 1<br>MILD	Grade 2<br>MODERATE	Grade 3<br>SEVERE	Grade 4<br>POTENTIALLY LIFE-THREATENING
**HEMATOLOGY**				
**HEMOGLOBIN**	9.5 g/dL–10.5 g/dL<br>OR<br>any decrease ≥2.5 g/dL	8.0 g/dL –9.4 g/dL<br>OR<br>any decrease ≥3.5 g/dL	6.5 g/dL–7.9 g/dL<br>OR<br>any decrease ≥4.5 g/dL	<6.5 g/dL
**ABSOLUTE NEUTROPHIL COUNT (ANC)**	1000 – 1500/mm^3	750 – 999/mm^3	500 –749/mm^3	<500/mm^3
**WHITE BLOOD CELL COUNT (WBC)**	11,500 – 13,000/mm^3	13,001– 15,000/mm^3	15,000 – 30,000/mm^3	>30,000/mm^3<br>OR<br><1,000/mm^3
**PLATELETS**	100,000 – 120,000/mm^3	99,999 – 75,000/mm^3	74,999 – 50,000/mm^3	<50,000/mm^3
**FIBRINOGEN**	100 mg/dL to <LLN<br>OR<br>>ULN to 600 mg/dL	50 - 99 mg/dL<br>OR<br>>600 mg/dL	<50 mg/dL<br>OR<br>associated with gross bleeding, or associated with disseminated coagulation	—
**PROTHROMBIN TIME (PT)**	>1.0 – 1.24 x ULN	>1.25 – 1.49 x ULN	>1.5 – 3.0 x ULN	>3.0 x ULN
**PARTIAL THROMBOPLASTIN TIME (PTT)**	>1.0 – 1.66 x ULN	>1.66 – 2.33 x ULN	>2.33 – 3.0 x ULN	>3.0 x ULN
***CHEMISTRY**				
**CREATINE PHOSPHOKINASE (CPK)**	≥4 x ULN	≥6 x ULN	≥10 x ULN	≥20 x ULN
**CREATININE**	>1.0 – 1.5 x ULN	>1.5 –2.0 x ULN	>2.0 – 6.0 x ULN	>6.0 x ULN
**SODIUM**<br>Hyponatremia<br>Hypernatremia	130 – 134 mEq/L<br>146 – 150 mEq/L	123 – 129 mEq/L<br>151 – 157 mEq/L	116 – 122 mEq/L<br>158 – 165 mEq/L	<116 mEq/L<br>>165 mEq/L
**POTASSIUM**<br>Hypokalemia<br>Hyperkalemia	3.2 – 3.4 mEq/L<br>5.0 – 5.5 mEq/L	3.0 – 3.1 mEq/L<br>5.6 – 6.0 mEq/L	2.5 – 2.9 mEq/L<br>6.1 – 6.5 mEq/L	<2.5 mEq/L<br>>6.6 mEq/L
**PHOSPHATE**<br>Hypophosphatemia	2.0 – 2.4 mg/dL	1.5 – 1.9 mg/dL	1.0 – 1.4 mg/dL	<1.0 mg/dL
**CALCIUM (corrected for albumin)**<br>Hypocalcemia<br>Hypercalcemia	7.8 – 8.4 mg/dL<br>10.6 – 11.5 mg/dL	7.0 – 7.7 mg/dL<br>11.6 – 12.5 mg/dL	6.1 – 6.9 mg/dL<br>12.6 – 13.5 mg/dL	<6.1 mg/dL<br>>13.5 mg/dL

Parameter	Grade 1<br>MILD	Grade 2<br>MODERATE	Grade 3<br>SEVERE	Grade 4<br>POTENTIALLY LIFE-THREATENING
**MAGNESIUM**<br>Hypomagnesemia	1.2 – 1.4 mEq/L	0.9 – 1.1 mEq/L	0.6 - 0.8 mEq/L	<0.6 mEq/L
**BILIRUBIN**<br>Hyperbilirubinemia	>1.0 – 1.5 x ULN	>1.5 – 2.5 x ULN	>2.5 – 5 x ULN	>5 x ULN
**GLUCOSE**<br>Hypoglycemia<br>Hyperglycemia<br>(nonfasting and no prior diabetes)	55 – 69 mg/dL<br>116 – 160 mg/dL	40 – 54 mg/dL<br>161 – 250 mg/dL	30 – 39 mg/dL<br>251 – 500 mg/dL	<30 mg/dL<br>>500 mg/dL
**TRIGLYCERIDES**	—	400 – 750 mg/dL	751 – 1200 mg/dL	>1200 mg/dL
**LIVER TRANSAMINASE**<br>ALT (SGPT)<br>AST (SGOT)<br>GGT<br>Alkaline Phosphatase	>1.25 – 3.0 x ULN<br>>1.25 – 2.5 x ULN<br>>1.25 – 2.5 x ULN<br>>1.25 – 2.5 x ULN	>3.0 – 5.0 x ULN<br>>2.5 – 5.0 x ULN<br>>2.5 – 5.0 x ULN<br>>2.5 – 5.0 x ULN	>5.0 – 10.0 x ULN<br>>5.0 – 10.0 x ULN<br>>5.0 – 10.0 x ULN<br>>5.0 – 10.0 x ULN	>10.0 x ULN<br>>10.0 x ULN<br>>10.0 x ULN<br>>10.0 x ULN
**PANCREATIC ENZYMES**<br>Amylase<br>Pancreatic amylase<br>Lipase	>1.0 – 1.5 x ULN<br>>1.0 – 1.5 x ULN<br>>1.0 – 1.5 x ULN	>1.5 – 2.0 x ULN<br>>1.5 – 2.0 x ULN<br>>1.5 – 2.0 x ULN	>2.0 – 5.0 x ULN<br>>2.0 – 5.0 x ULN<br>>2.0 – 5.0 x ULN	>5.0 x ULN<br>>5.0 x ULN<br>>5.0 x ULN
* The normal range for a laboratory parameter may vary over time. A laboratory value will not be categorized as a Grade 1 unless the value is outside of the normal range for the laboratory conducting the test at the time the test is completed.				
**CARDIOVASCULAR**				
**CARDIAC ARRHYTHMIA**	—	asymptomatic; transient dysrhythmia; no therapy required	recurrent/persistent dysrhythmia; symptomatic therapy required	unstable dysrhythmia, hospitalization and therapy required
**HYPERTENSION**	transient, increase >20 mm Hg diastolic BP; no therapy required	recurrent; chronic increase >20 mm Hg diastolic BP; therapy required	acute therapy required; outpatient or hospitalization possible	hospitalization required, or end organ damage
**HYPOTENSION**	transient orthostatic hypotension with heart rate increased by >20 beats/min or decreased by <10 mm Hg systolic BP, no therapy required	symptoms or BP decreased by <20 mm Hg systolic, correctable with oral fluid therapy	IV fluids required, or hospitalization	mean arterial pressure <60 mm Hg, or end organ damage, or shock, vasopressor therapy required
**PERICARDITIS**	minimal effusion	mild/moderate asymptomatic effusion, no therapy	symptomatic effusion, pain, EKG changes	tamponade or pericardiocentesis or surgery required
**HEMORRHAGE, BLOOD LOSS**	—	mildly symptomatic, no therapy required	gross blood loss or 1 – 2 units transfused	massive blood loss or >2 units transfused

Parameter	Grade 1 MILD	Grade 2 MODERATE	Grade 3 SEVERE	Grade 4 POTENTIALLY LIFE-THREATENING
**GASTROINTESTINAL**				
**VOMITING**	mild or transient; 2 – 3 episodes per day or mild vomiting lasting <1 week	moderate or persistent; 4 – 5 episodes per day; or vomiting lasting ≥1 week	severe vomiting of all food/fluids in 24 hours or orthostatic hypotension or IV therapy required	hypotensive shock or hospitalization required for IV therapy
**DIARRHEA**	mild or transient; 3 - 4 loose stools per day or mild diarrhea lasting less than 1 week	moderate or persistent; 5 - 10 loose stools per day or diarrhea lasting ≥1 week	>10 loose stools/day bloody diarrhea; or orthostatic hypotension or electrolyte imbalance, >2 L IV fluid required	hypotensive shock or severe electrolyte imbalance
**ORAL DISCOMFORT/DYSPHAGIA**	mild discomfort, no difficulty swallowing	difficulty swallowing but able to eat and drink	unable to swallow solids	unable to drink fluids; IV fluids required
**CONSTIPATION**	—	moderate abdominal pain 78 hours with impaction, require output prescription	requiring disimpaction or hospital treatment	distention with vomiting or obstipation
**RESPIRATORY**				
**COUGH (FOR AEROSOL STUDIES)**	transient; no therapy	treatment-associated cough; inhaled bronchodilator	uncontrolled cough; systemic therapy required	—
**BRONCHOSPASM ACUTE**	transient; no therapy; FEV1 or peak flow reduced to 70 - <80%	therapy required; normalizes with bronchodilator; FEV1 or peak flow 50 - 69%	no normalization with bronchodilator; FEV1 or peak flow 25 – 49%, retractions	cyanosis; FEV1 or peak flow <25% or intubated
**DYSPNEA**	dyspnea on exertion	dyspnea with normal activity	dyspnea at rest	dyspnea requiring 0_2 therapy
**NEUROLOGIC**				
**NEUROCEREBELLAR**	slight incoordination or dysdiadochokinesia	intention tremor or dysmetria or slurred speech or nystagmus	ataxia requiring assistance to walk or arm incoordination interfering with ADLs	unable to stand
**NEUROPSYCHIATRY/MOOD**	—	—	severe mood changes requiring medical intervention; suicidal ideation	acute psychosis requiring hospitalization; suicidal gesture/attempt
**PARESTHESIA (BURNING, TINGLING, ETC.)**	mild discomfort; no therapy required	moderate discomfort; non-narcotic analgesia required	severe discomfort; or narcotic analgesia required with symptomatic improvement	incapacitating; or not responsive to narcotic analgesia

Parameter	Grade 1<br>MILD	Grade 2<br>MODERATE	Grade 3<br>SEVERE	Grade 4<br>POTENTIALLY LIFE-THREATENING
**NEUROMOTOR**	mild weakness in muscle of feet, but able to walk; and/or mild increase or decrease in reflexes	moderate weakness in feet (unable to walk on heels and/or toes), mild weakness in hands, still able to do most hand tasks and/or loss of previously present reflex or development of hyperreflexia and/or unable to do deep knee bends due to weakness	marked distal weakness (unable to dorsiflex toes or foot drop), and moderate proximal weakness, e.g., in hands interfering with ADLs and/or requiring assistance to walk and/or unable to rise from chair unassisted	confined to bed or wheelchair because of muscle weakness
**NEUROSENSORY**	mild impairment (decreased sensation, e.g., vibratory, pinprick, hot/cold in great toes) in focal area or symmetrical distribution	moderate impairment (moderate decreased sensation, e.g., vibratory, pinprick, hot/cold to ankles) and/or joint position or mild impairment that is not symmetrical	severe impairment (decreased or loss of sensation to knees or wrists) or loss of sensation of at least moderate degree in multiple different body areas (i.e., upper and lower extremities)	sensory loss involves limbs and trunk
**URINALYSIS**				
**PROTEINURIA** Random urine<br>24 hour urine	1+<br>200 mg – 1 g loss/day or <0.3% or <3 g/L	2+, 3+<br>>1 – 2 g loss/day or 0.3% - 1.0% or 3 – 10 g/L	4+<br>>2 – 3.5 g loss/day or >1.0% or >10 g/L	nephrotic syndrome<br>nephrotic syndrome or >3.5 g loss/day
**HEMATURIA**<br>(in the absence of vaginal bleeding)	microscopic only, 6-10 rbc/hpf	>10 rbc/hpf	gross, with or without clots; or RBC casts	obstructive, or transfusion required
**MISCELLANEOUS**				
**ALLERGIC REACTION**	pruritus without rash at injection site	localized urticaria at injection site	generalized urticaria angioedema	anaphylaxis
**ACTIVITIES OF DAILY LIVING**	normal activity reduced <48 hours	normal activity reduced 25 – 50% >48 hours	normal activity reduced >50%; cannot work >48 hours	unable to care for self
**EYE**	—	mild pain, visual changes, conjunctival erythema, abnormal slit lamp	loss of vision, clinically diagnosed uveitis, moderate to severe pain, glaucoma	—
