# Supplementary material for: A Single Dose of the DENV-1 Candidate Vaccine rDEN1Δ30 Is Strongly Immunogenic and Induces Resistance to a Second Dose in a Randomized Trial
Source: PLoS Negl Trop Dis. 2011 Aug 2;5(8):e1267. doi: 10.1371/journal.pntd.0001267 (PMC3149013; doi:10.1371/journal.pntd.0001267)
Supplement: Text S1 — IRB approvals. (PDF) [file pntd.0001267.s003.pdf]

WIRB®

(360) 252-2500
1-800-562-4789
FAX: (360) 252-2498

Western Institutional Review Board ®

Western International Review Board®

3535 SEVENTH AVENUE, SW, OLYMPIA, WA 98502-5010
P.O. BOX 12029, OLYMPIA, WA 98508-2029

Certificate of Approval

THE FOLLOWING WERE APPROVED:

INVESTIGATOR: Anna P. Durbin M.D.
Center for Immunization Research
Hampton House, Room 117
624 North Broadway
Baltimore, Maryland 21205

BOARD ACTION DATED: 05/02/2007
PANEL: 3
STUDY APPROVAL EXPIRES: 05/02/2008
STUDY NUM: 1089537
WIRB PRO NUM: 20070718
INVEST NUM: 115264
WO NUM: 1-427640-1

SPONSOR: National Institutes of Health/NIAID/RCHSPB
PROTOCOL NUM: 229
AMD. PRO. NUM:
TITLE:
Safety and Immunogenicity of a 2-Dose Regimen of rDEN1Δ30 Dengue Serotype 1 Vaccine with Boosting at 4 versus 6 Months

APPROVAL INCLUDES:

Investigator
Protocol (03-08-2007) Version 1.0
Consent Form - Skin Biopsy [IN0]
Consent Form [IN0]
Dengue Comprehension Quiz #4426826.0 - As Submitted
Dengue Vaccine Temperature Diary #4439230.0 - As Submitted
How to Stop Mosquitos #4437575.0 - As Submitted
Initial Contact #4426823.0 - As Submitted
Medical History Form #4426825.0 - As Submitted
Missed Appointment Letter #4426831.0 - As Modified

WIRB APPROVAL IS GRANTED SUBJECT TO:

The Board directed that persons who are unable to read are not allowed to consent for themselves or others to participate in this study.

The Board requires that all subjects must be able to consent for themselves to be enrolled in this study.

IF YOU HAVE ANY QUESTIONS, CONTACT WIRB AT 1-800-562-4789
This is to certify that the information contained herein is true and correct as reflected in the records of the Western Institutional Review Board (WIRB). WE CERTIFY THAT WIRB IS IN FULL COMPLIANCE WITH GOOD CLINICAL PRACTICES AS DEFINED UNDER THE U.S. FOOD AND DRUG ADMINISTRATION (FDA) REGULATIONS AND THE INTERNATIONAL CONFERENCE ON HARMONISATION (ICH) GUIDELINES.

Association for the Accreditation of Human Research Protection Programs, Inc. — Full AAHRPP Accreditation

Theodore D. Schultz, J.D., Chairman

5/4/2007
(Date)

This document electronically reviewed and approved by Orive, Otto on 5/4/2007 6:54:46AM PST. For more information call Client Services at 1-360-252-2500

Copyright © 2006 Western Institutional Review Board, Inc. All rights reserved.

APPROVAL INCLUDES Cont:

Missed Appointment Letter #4426832.0 - As Submitted

Pregnancy Prevention Assessment #4426830.0 - As Submitted

Registration Form #4426824.0 - As Submitted

Treatment Notification Letter #4426833.0 - As Submitted

Visit Schedule - 4 Month Cohort #4426834.0 - As Modifed

Visit Schedule - 6 Month Cohort #4426835.0 - As Modified

ALL WIRB APPROVED INVESTIGATORS MUST COMPLY WITH THE FOLLOWING:

1. Conduct the research in accordance with the protocol, applicable laws and regulations, and the principles of research ethics as set forth in the Belmont Report.

2. Unless consent has been waived, conduct the informed consent process without coercion or undue influence, and provide the potential subject sufficient opportunity to consider whether or not to participate.

    a. Use only the most current consent form bearing the WIRB "APPROVED" stamp.

    b. Provide non-English speaking subjects with a certified translation of the approved consent form in the subject's first language. The translation must be approved by WIRB.

    c. Obtain pre-approval from WIRB for use of recruitment materials and other materials provided to subjects.

3. Obtain pre-approval from WIRB for any planned deviations that could adversely affect the safety or welfare of subjects, or the integrity of the research data and any changes in the research activity. The only exception is when changes are necessary to eliminate apparent immediate hazards to subjects. Immediately report to WIRB any such emergency changes implemented.

4. Promptly report to WIRB any new information that may adversely affect the safety of the subjects or the conduct of the trial.

    a. Report to WIRB all adverse events that are unanticipated and possibly related, within 10 days of the investigator becoming aware of them.

    b. Promptly report to WIRB other unanticipated problems involving risks to human subjects or others. These events do not readily fit the formal definition of Adverse Event, but could impact human subject safety and/or rights. Examples include theft of a computer containing private identifiable subject information, or study staff getting ill from inhaling a study agent.

    c. Provide reports to WIRB concerning the progress of the research, when requested.

5. Report to WIRB any unplanned protocol variance that could adversely affect the safety or welfare of subjects, or the integrity of the research data, within 10 days of becoming aware of the variance. Other unplanned variances may be recorded on a log and submitted with continuing review reports.

Federal regulations require that WIRB conduct continuing review of approved research. You will receive Continuing Review Report forms from WIRB. These reports must be returned even though your study may not have started.

DISTRIBUTION OF COPIES:

Contact	Company Name
Alice B. Chase	The Johns Hopkins University Bloomberg School of Public Health
Anna P. Durbin M.D.	The Johns Hopkins University Bloomberg School of Public Health
Loretta Polite C.R.A.	The Johns Hopkins University Bloomberg School of Public Health

SITES: If the PI has an obligation to use another IRB for any site listed below and has not submitted a written statement from the other IRB acknowledging WIRB's review of this research, please contact WIRB's Client Services department.

Address

Center for Immunization Research, Hampton House, Room 117, 624 North Broadway, Baltimore, Maryland 21205

Center for Immunization Research, Suite 305, 2112 F Street NW, Washington, District of Columbia 20037

Copyright © 2006 Western Institutional Review Board, Inc. All rights reserved.

WIRB®

(360) 252-2500
1-800-562-4789
FAX: (360) 252-2498

Western Institutional Review Board ®

3535 SEVENTH AVENUE, SW, OLYMPIA, WA 98502-5010
P.O. BOX 12029, OLYMPIA, WA 98508-2029

Certificate of Approval

THE FOLLOWING WERE APPROVED:

INVESTIGATOR: Anna P. Durbin M.D.
Center for Immunization Research
Hampton House, Room 117
624 North Broadway
Baltimore, Maryland 21205

BOARD ACTION DATE: 4/7/2010
PANEL: 3
STUDY APPROVAL EXPIRES: 5/2/2011
STUDY NUM: 1089537
WIRB PRO NUM: 20070718
INVEST NUM: 115264
WO NUM: 1-604372-1
CONTINUING REVIEW: Annually
SITE STATUS REPORTING: Annually

SPONSOR: National Institutes of Health/NIAID/RCHSPB
PROTOCOL NUM: 229
AMD. PRO. NUM:
TITLE:
Safety and Immunogenicity of a 2-Dose Regimen of rDEN1Delta30 Dengue Serotype 1 Vaccine with Boosting at 4 versus 6 Months

APPROVAL INCLUDES:
Study and Investigator for an additional continuing review period. This approval expires on the date noted above.

WIRB APPROVAL IS GRANTED SUBJECT TO:

IF YOU HAVE ANY QUESTIONS, CONTACT WIRB AT 1-800-562-4789
This is to certify that the information contained herein is true and correct as reflected in the records of the Western Institutional Review Board (WIRB), OHRP/FDA parent organization number IORG 0000432, IRB registration number IRB00000533. WE CERTIFY THAT WIRB IS IN FULL COMPLIANCE WITH GOOD CLINICAL PRACTICES AS DEFINED UNDER THE U.S. FOOD AND DRUG ADMINISTRATION (FDA) REGULATIONS AND THE INTERNATIONAL CONFERENCE ON HARMONISATION (ICH) GUIDELINES.

Theodore D. Schultz, J.D., Chairman

4/21/2010
(Date)

This document electronically reviewed and approved by Ennever, John on 4/21/2010 12:44:39 AM PST. For more information call Client Services at 1-360-252-2500

Copyright © 2008 Western Institutional Review Board, Inc. All rights reserved.

WIRB HAS APPROVED THE FOLLOWING LOCATIONS TO BE USED IN THE RESEARCH:

- Johns Hopkins Bloomberg School of Public Health, Center for Immunization Research, Hampton House, Room 117, 624 North Broadway, Baltimore, Maryland 21205
- Johns Hopkins Bloomberg School of Public Health, Center for Immunization Research, Suite 305, 2112 F Street NW, Washington, District of Columbia 20037

If the PI has an obligation to use another IRB for any site listed above and has not submitted a written statement from the other IRB acknowledging WIRB's review of this research, please contact WIRB's Client Services department.

ALL WIRB APPROVED INVESTIGATORS MUST COMPLY WITH THE FOLLOWING:

1. Conduct the research in accordance with the protocol, applicable laws and regulations, and the principles of research ethics as set forth in the Belmont Report.

2. Although a participant is not obliged to give his or her reasons for withdrawing prematurely from the clinical trial, the investigator should make a reasonable effort to ascertain the reason, while fully respecting the participant’s rights.

3. Unless consent has been waived, conduct the informed consent process without coercion or undue influence, and provide the potential subject sufficient opportunity to consider whether or not to participate. (Due to the unique circumstances of research conducted at international sites outside the United States and Canada where WIRB approved materials are translated into the local language, the following requirements regarding consent forms bearing the WIRB approval stamp and regarding certification of translations are not applicable.)

    a. Use only the most current consent form bearing the WIRB "APPROVED" stamp.

    b. Provide non-English speaking subjects with a certified translation of the approved consent form in the subject's first language. The translation must be approved by WIRB.

    c. Obtain pre-approval from WIRB for use of recruitment materials and other materials provided to subjects.

4. Obtain pre-approval from WIRB for changes in research.

5. Obtain pre-approval from WIRB for any planned deviations that could adversely affect the rights, safety or welfare of subjects, or the integrity of the research data and any changes in the research activity. The only exception is when changes are necessary to eliminate apparent immediate hazards to subjects. Deviations necessary to eliminate apparent immediate hazards to the human subjects should be reported within 10 days.

6. Promptly report to WIRB all unanticipated problems (adverse events, protocol deviations and violations and other problems) that meet all of the following criteria:

    a. Unexpected (in terms of nature, severity or frequency);

    b. Related or possibly related to participation in the research; and

    c. Suggests that the research places subjects or others at a greater risk of harm than was previously known or recognized.

    Please go to www.wirb.com for complete definitions and forms for reporting.

7. Provide reports to WIRB concerning the progress of the research, when requested.

8. Ensure that prior to performing study-related duties, each member of the research study team has had training in the protection of human subjects appropriate to the processes required in the approved protocol.

Federal regulations require that WIRB conduct continuing review of approved research. You will receive Continuing Review Report forms from WIRB. These reports must be returned even though your study may not have started.

Copyright © 2008 Western Institutional Review Board, Inc. All rights reserved.

DISTRIBUTION OF COPIES:

Contact	Company Name
Anna P. Durbin M.D.	The Johns Hopkins University Bloomberg School of Public Health
Joan C. Pettit J.D., MA	The Johns Hopkins University Bloomberg School of Public Health
Eunice Kagucia	The Johns Hopkins University Bloomberg School of Public Health

Copyright © 2008 Western Institutional Review Board, Inc. All rights reserved.
